# Deciding for others alters metacognition leading to responsibility aversion

**DOI:** 10.1126/sciadv.ady0441

**Published:** 2026-02-25

**Authors:** Sherry Dongqi Bao, Micah G. Edelson, Todd A. Hare

**Affiliations:** Zurich Center for Neuroeconomics, Department of Economics, University of Zurich, Zurich, Switzerland.

## Abstract

People are often faced with choices whose outcomes will affect other individuals in addition to themselves. Being responsible for deciding on behalf of others alters choice behavior and increases delegation rates in decisions involving risk and ambiguity. However, it is unknown whether the influence of social responsibility on decision-making acts primarily or directly on risk, loss, and ambiguity attitudes versus more general aspects of cognition. We report a series of experiments on objective magnitude judgments that demonstrate that the influences of responsibility on cognition and behavior extend beyond risk or ambiguity and act at the metacognitive level. Specifically, responsibility for others changes metacognitive biases, leading to a decrease in decision confidence without affecting choice accuracy. Last, we propose and empirically test a normative computational framework based on decision confidence that can explain decisions to assume or delegate responsibility for others without needing to assume changes in risk preferences.

## Teaser

Making decisions on behalf of other people reduces decision confidence, which leads to responsibility aversion.

## INTRODUCTION

We live in highly complex social environments and make many of our most important decisions in the context of social interactions (*1*). A particularly relevant aspect of social decision-making is the taking of responsibility for others, a common scenario in daily life with widespread and lasting impacts on both decision-makers and those affected by their choice outcomes (*2*). For example, decisions made by parents affect their children, those by teachers influence their students, and those by business or government leaders have consequences for their group members and society at large. Responsibility for others has been shown to change whether and how people make such decisions. Here, we elucidate the influence of social responsibility on metacognitive evaluations of decision certainty and the consequences of these metacognitive effects on behavior.

Prior research has predominantly focused on how social responsibility affects risk-related decisions. Several studies have reported that decision-makers exhibit greater risk aversion (*3*–*6*), loss aversion, and consistency in their decisions when their choices affect others (*4*). In contrast, separate studies have shown that responsibility-induced changes in risk or loss preferences depend on choice sets and that responsibility may even increase risk seeking in some cases [e.g., in the loss domain or with small-probability gains (*7*, *8*)]. Furthermore, other work found that changes in risk or ambiguity preferences could not adequately explain the effects of social responsibility on decision-making (*9*).

Thus, despite the longstanding interest in decision-making under social responsibility, the cognitive mechanisms at play when individuals are faced with making decisions for others remain incompletely understood. Is explicit risk (i.e., aleatoric uncertainty) a necessary condition for social responsibility to affect decision-making? Are risk preferences the direct and/or only channel through which these effects occur? Until now, additional or alternative mechanisms beyond risk have received little attention.

We hypothesized that a change in metacognitive certainty is a key, but as yet unexplored, mechanism through which responsibility for others alters decision-making. Metacognitive certainty, defined as the degree of confidence a decision-maker has in the accuracy of their choice or proposition, influences behaviors ranging from fundamental judgments in perception (*10*) and memory (*11*), to valuation (*12*), risky choices (*13*), and information-seeking (*14*–*17*). We know that metacognitive processes play a fundamental role in shaping healthy and disordered social behavior (*18*–*20*). However, little is known about the relationship between responsibility and metacognitive certainty. One clue comes from previous work showing that participants faced with social responsibility were more likely to delegate to a group vote for harder decisions (i.e., smaller value differences), which suggests that decision certainty is important in this process (*9*). Regrettably, previous studies on responsibility have not measured decision certainty and thus cannot speak directly to the potential for responsibility to induce metacognitive effects. Moreover, the normative relationship between a potential decision-maker’s certainty and their willingness or aversion to make decisions on behalf of others has not yet been developed or empirically tested. Thus, there remains a critical gap in our understanding of the potential link between metacognitive decision certainty and responsibility aversion as well as other aspects of social responsibility.

Our current work bridges these gaps in understanding decision-making with social responsibility. We achieve this by directly measuring decision certainty through confidence ratings in a series of magnitude judgment tasks. This decision task allows for objective measures of choice accuracy, enabling a detailed investigation of the role of metacognition and its accuracy in decision-making under social responsibility. Notably, the magnitude judgments did not involve risk, allowing us to test whether responsibility for others affects cognitive processes beyond this factor. We tested obligatory decision-making (i.e., scenarios with no option to delegate or avoid deciding) under various conditions: those with either limited or unlimited decision evidence, those with or without feedback on performance accuracy, and those in social and nonsocial contexts. This comprehensive approach allowed us to determine the cognitive mechanisms involved and isolate the specific effects of social responsibility. In addition, we developed a normative framework for responsibility aversion through the lens of metacognition. We empirically tested this framework by quantifying behavior in a second choice context in which decision-makers had the option to delegate the choice and consequently the responsibility for others. Overall, we find that changes in decision confidence can explain critical aspects of how social responsibility influences decision-making.

## RESULTS

**Fig. 1.**
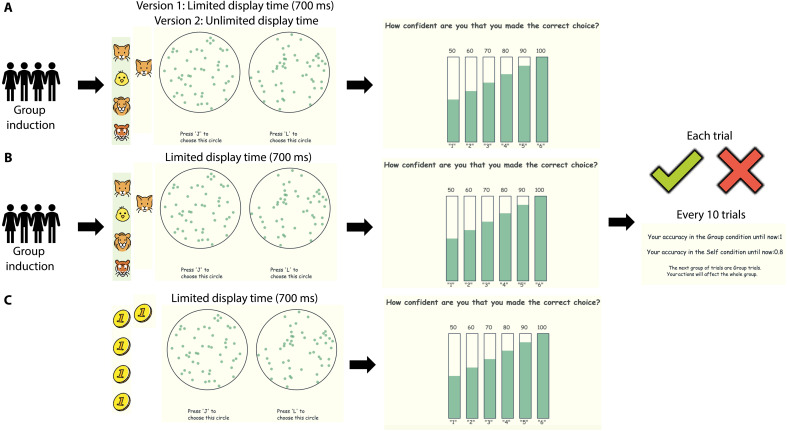
The graphics outline key features of different versions of the forced-choice behavioral task. Participants must decide which of the two circles contain more dots. The payoff for accurate responses affects either themselves or everyone in their group (including themselves). The number of icons on the screen indicate whom the payoff applies to. For all social versions of this task shown in rows (A) and (B), the procedure is the same. Participants first go through a group induction phase before completing the task. (**A**) Experiments without feedback on accuracy: In version 1, the stimuli are displayed for 700 ms, whereas in version 2, the stimuli are displayed for an unlimited amount of time until participants make a decision. In both versions, participants indicate their confidence after making the decision. (**B**) Experiment with feedback on accuracy: The stimuli are displayed for 700 ms; after participants make their decision and rate their confidence, feedback about the correctness of their decision is shown. In addition, there is a feedback summary for each condition after every 10 trials. (**C**) The nonsocial version of the task includes a low-stakes condition and a high-stakes condition in which the potential payoff is four times that of the low-stakes trials.

We investigated whether human behavior differs in a simple decision task that required participants to determine which of two circles contained more dots under conditions in which the person’s decision accuracy affects (i) only their own payoff (Self), or (ii) their own plus others’ payoffs (Group). After making the decision, participants reported their confidence in being correct using an incentive-compatible mechanism that is robust to risk preferences (*21*–*24*). The task paradigm is shown in Fig. 1. Specifically, we compared participants’ accuracy, confidence ratings, and response times (RTs) under these two conditions. We ran multiple versions of the experiment to better elucidate the relationship between confidence and social responsibility. First, we focus on the results from the forced-choice versions of this task in which participants were obliged to make the decision themselves. These experiments demonstrate that social responsibility decreases confidence. Later, we present the findings from a version of the task in which participants could delegate their decision power to others to show that changes in confidence are sufficient to explain the observed increase in delegation proportions under responsibility for others.

### The impact of social responsibility on decision accuracy, confidence, and RTs

**Fig. 2.**
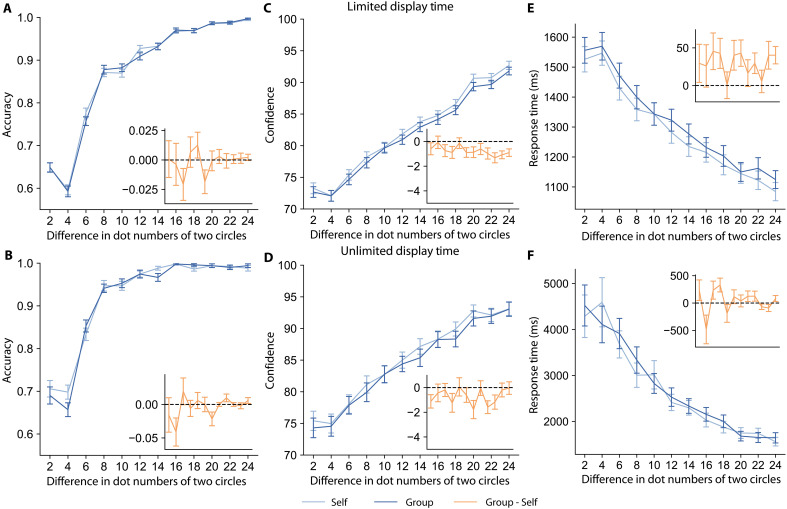
Social responsibility influences confidence and decision RTs but not accuracy. The *y* axis of each plot shows the mean accuracy (left), confidence (middle), or decision RT (right) across various stimulus strengths along the *x* axis for the experiment with limited stimulus display time (top row) and unlimited stimulus display time (bottom row). Trials from the Group condition are shown in dark blue, and Self trials are in light blue. The orange inset of each plot shows the average difference (Group minus Self) for the corresponding measurement of each trial. Error bars indicate the SEM across participants. (**A** and **B**) There is no significant difference in accuracy between the Group and Self conditions regardless of stimulus display time. (**C** and **D**) Participants have lower confidence when taking responsibility for others compared to deciding for themselves alone in both limited-display and unlimited-display time experiments. (**E** and **F**) Participants respond slower when they are responsible for others compared to deciding only for themselves in both task versions.

Participants reported lower confidence in their decisions when responsible for others, despite being equally accurate in making decisions on behalf of themselves alone (Self trials) or themselves plus others (Group trials). Figure 2 (A and B) illustrates the average accuracy across participants at each stimulus strength level in both conditions for these two experiments. We use several mixed-effects Bayesian regressions to examine accuracy, confidence, and RTs, and to facilitate reference back to the respective regression equations in the Materials and Methods, we number the coefficients of interest (β) from these models according to the equation number followed by the serial position within the equation (e.g., $\beta_{1.1}$ comes from Eq. 1). The logistic regression for accuracy shows that as the difference in the number of dots increases, participants are more accurate in their choices {Eq. 1; main effect of stimulus strength in limited-display experiment: $\beta_{1.2}=1.44$, 95% credible interval (CI) = [1.39, 1.49], $P_{\text{MCMC}}<0.001$; in unlimited-display experiment: $\beta_{1.2}=1.85$, 95% CI = [1.73, 1.97], $P_{\text{MCMC}}<0.001$}. As expected, accuracy is higher when the dots are shown for an unlimited time compared to a limited time (Eq. 4; main effect of experiment type: $\beta_{4.3}=0.54$, 95% CI = [0.46, 0.61], $P_{\text{MCMC}}<0.001$). Critically, there is no significant difference in accuracy between the Group and Self conditions, either when the stimulus is displayed for a limited time (main effect of condition: $\beta_{1.1}=0.02$, 95% CI = [−0.06, 0.09], $P_{\text{MCMC}}=0.34$) or an unlimited time (main effect of condition: $\beta_{1.1}=0.06$, 95% CI = [−0.08, 0.20], $P_{\text{MCMC}}=0.2$).

In contrast to decision accuracy, decision confidence was significantly different under responsibility for others. Figure 2 (C and D) shows that participants’ confidence trend mirrors their accuracy trend across stimulus strengths, confidence increases with greater stimulus strength (Eq. 2; main effect of stimulus strength in limited-display experiment: $\beta_{2.2}=0.41$, 95% CI= [0.40, 0.42], $P_{\text{MCMC}}<0.001$; in unlimited-display experiment: $\beta_{2.2}=0.42$, 95% CI = [0.41, 0.44], $P_{\text{MCMC}}<0.001$), and confidence is higher, on average, when the stimulus is shown for an unlimited time (Eq. 5; main effect of experiment type: $\beta_{5.3}=0.05$, 95% CI = [0.04, 0.06], $P_{\text{MCMC}}<0.001$). However, participants have lower confidence when taking responsibility for others compared to when deciding for themselves alone, both in the limited (main effect of condition: $\beta_{2.1}=-0.05$, 95% CI = [−0.07, −0.03], $P_{\text{MCMC}}<0.001$) and unlimited (main effect of condition: $\beta_{2.1}=-0.05$, 95% CI = [−0.09, 0.003], $P_{\text{MCMC}}=0.02$) stimulus display experiments. This indicates that responsibility for others decreases decision confidence, regardless of whether one could theoretically sample more evidence to reach higher certainty for their decisions in the unlimited display time experiment. Note that the change in confidence induced by social responsibility did not vary as a function of epistemic uncertainty (i.e., in the difference in number of dots between the two circles) in the magnitude judgment task (Fig. 2, C and D, and table S1). Lastly, decreases in confidence were correlated with slower decision RTs in the limited and unlimited stimulus display time experiments (fig. S1 and Supplementary Text). However, the association between confidence and RT effects was not found in a version of the experiment that provided feedback on performance (details below), indicating that the two effects are at least partially independent.

Decision RTs were slower under responsibility for others. Figure 2 (E and F) shows that, when trials are easier, people tend to have shorter decision RTs (Eq. 3; main effect of stimulus strength on the natural logarithm of RTs in limited-display experiment: $\beta_{3.2}=-0.099$, 95% CI = [−0.10, −0.095], $P_{\text{MCMC}}<0.001$; in unlimited-display experiment: $\beta_{3.2}=-0.26$, 95% CI = [−0.27, −0.25], $P_{\text{MCMC}}<0.001$). Critically, there is also a main effect of responsibility such that participants spent a longer time responding when they were responsible for others compared to deciding only for themselves, both in limited-display (main effect of condition: $\beta_{3.1}=0.020$, 95% CI = [0.011, 0.028], $P_{\text{MCMC}}<0.001$) and unlimited-display (main effect of condition $\beta_{3.1}=0.023$, 95% CI = [−0.0047, 0.0501], $P_{\text{MCMC}}=0.05$) experiments. Recall that participants achieved similar accuracy in the Group and Self conditions, and thus these differences in decision RTs do not represent a speed-accuracy trade-off. Lastly, in addition to decision RTs, social responsibility also led to slower trial initiation times and confidence rating times (fig. S6 and Supplementary Text).

### Decision mechanisms underlying behavior changes with social responsibility

Given its distinct effects on accuracy and decision RTs, we tested how responsibility for others changed the parameters of a decision framework that jointly accounts for both measures. Regrettably, there is no widely agreed upon modeling framework to decision accuracy, RT, and confidence simultaneously. Thus, we fit the data to a drift diffusion model (DDM) with collapsing bounds to explain the cognitive mechanism underlying accuracy and RT patterns alone, and address confidence separately below. We used a DDM with collapsing bound instead of fixed bound because past work showed that collapsing bounds better approximate the optimal process and explain the empirical data better when participants complete a series of trials with varying difficulty levels—as is the case in our experiments (*25*). Note, however, that our findings generalize to alternative sequential sampling model specifications (fig. S7 and the Supplementary Text).

We fitted DDMs to the data in Group and Self conditions separately (see table S2 for a summary of the fitted parameters). After fitting, we conducted parameter sensitivity analyses to test whether changes in specific parameters were sufficient to reproduce the effects observed in the empirical data. We used each fitted parameter from the Group condition separately (bound, drift, nondecision time, and starting-point bias) combined with remaining fitted parameters from the Self condition to simulate data; afterward, we computed the same generalized linear mixed-effects regression models fit to the empirical data using the simulations to test how well specific parameter changes could account for the observed effects of responsibility for others on RT together with a consistent accuracy across conditions.

**Fig. 3.**
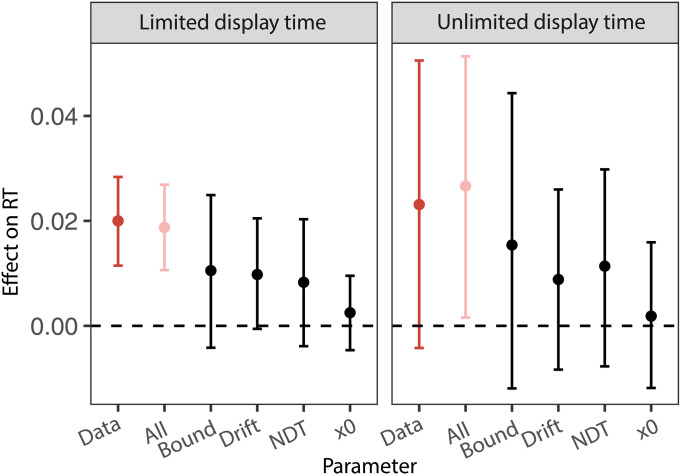
Social responsibility affects RTs through changes in multiple DDM parameters. The error bar plots show the result of a parameter sensitivity analysis that tested whether responsibility-induced changes to specific subsets of the parameters in the DDM can reproduce the effects observed in the behavioral data. Here, the effect of interest on the *y* axis is the influence of the social responsibility on RT, estimated from the generalized linear mixed model in Eq. 3. The *x* axis shows the effect in the empirical data, “Data,” and simulations from the DDM under different specifications. The simulations are labeled as follows: “All” includes changes in all fitted parameters; “Bound” uses the bound parameters fit separately to each condition and keeps other parameters fixed across conditions; similarly “Drift,” “NDT,” and “x0” include only changes in the drift rate, nondecision time, or starting-point bias, respectively, while keeping all other parameters fixed. Error bars represent the 95% CI for the fitted main effect of the Group condition on RT.

As shown in Fig. 3 and fig. S2, the simulations using all fitted parameters from the corresponding conditions can recreate the effect of social responsibility on RT while maintaining consistent accuracy, but no single parameter change can generate the observed pattern between the two conditions. Across both stimulus display times, the longer decision RT with social responsibility is a combined effect of higher bound, smaller drift rate, and longer nondecision time. This suggests that the effect of social responsibility on the cognitive mechanism underlying the perceptual processes in our task is multifaceted (not captured by a single parameter), and people may have both lower processing speed and more caution, which is captured by the DDM (drift rate and boundary separation parameters) (*26*), together with slower encoding, response execution, or other processes not directly parameterized by the DDM (nondecision time) (*27*).

### Characterizing metacognitive change

Next, we examined the metacognitive processes underlying the decrease in confidence during Group trials. To gain further insight into the changes in the underlying metacognitive processes, we applied an established metacognitive model (*28*) to our data to distinguish between responsibility effects on metacognitive noise and biases (see table S3 for a summary of the fitted parameters). Increases in metacognitive noise or efficiency would render confidence ratings more indifferent with respect to the level of sensory evidence, while changes to metacognitive biases would produce systematic discrepancies between objective performance and confidence (*28*).

**Fig. 4.**
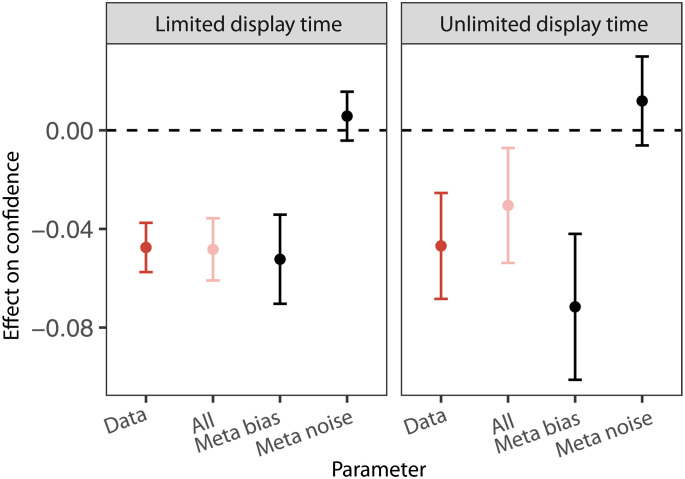
Social responsibility changes confidence primarily through effects on metacognitive bias. The error bar plots show the result of a parameter sensitivity analysis that tested whether responsibility-induced changes to specific subsets of the parameters in the metacognitive model can reproduce the effects observed in the behavioral data. Here, the effect of interest on the *y* axis is the influence of the Group condition on confidence, estimated from the generalized linear mixed model in Eq. 2. The *x* axis shows the effect in the empirical data, data, and simulations from the metacognitive model under different specifications. The simulations are labeled as follows: All includes changes in all fitted parameters; “Meta bias” uses metacognitive bias parameters fit separately to each condition and keeps other parameters fixed across conditions; and “Meta noise” includes only changes in the metacognitive noise parameter, keeping all others fixed. Error bars represent the 95% CI for the fitted main effect of the Group condition on confidence.

Once again, we used parameter sensitivity analysis to measure how the interdependent contributions of the metacognitive model parameters related to the patterns observed in participants’ behavior. This analysis of the responsibility effects indicated that a change in metacognitive bias, rather than noise, is the main contributor to the lower confidence with social responsibility (Fig. 4). Note that participants were also underconfident in Self trials compared to the simulated behavior of unbiased agents (limited display experiment: main effect of “simulation using fitted parameters in Self condition” compared to “simulation using no metacognitive bias in Self condition”: $\beta_{7.1}=-0.32$, 95% CI = [−0.40, −0.24], $P_{\text{MCMC}}<0.001$; unlimited display task: $\beta_{7.1}=-0.30$, 95% CI = [−0.44, −0.17], $P_{\text{MCMC}}<0.001$). Thus, responsibility for others exaggerated participants’ metacognitive biases, making them even less well calibrated than when deciding for themselves alone.

### Does feedback about decision accuracy close the confidence gap for responsibility?

It has been shown that feedback about performance accuracy can reduce metacognitive biases (*29*). Changes in metacognitive bias explain most of the confidence change under responsibility, and accuracy is not affected by responsibility for others. Therefore, we hypothesized that providing feedback about accuracy could diminish the effect of social responsibility on confidence. Such a feedback treatment helps to rule out the possibility that the lower confidence observed in the Group condition was due to participants’ erroneous belief that their performance was inferior with responsibility for others. To that end, we ran an experiment with a separate sample of 108 participants in which we provided feedback on their accuracy after they made the choice and rated their confidence on each trial and additionally as a summary of each condition after every 10 trials, which would directly show people that their performance in the two conditions is similar. This experiment had a limited display time of 700 ms (i.e., the same as in the limited-display time experiment that did not provide feedback).

**Fig. 5.**
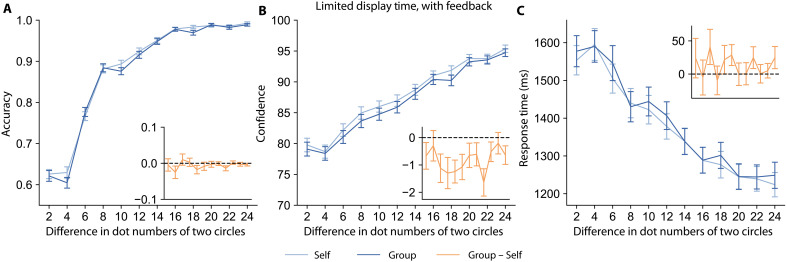
Social responsibility reduces confidence even if performance feedback is given. The *y* axis of each plot shows the mean accuracy (left), confidence (middle), or RT (right) across various stimulus strengths along the *x* axis. Trials from the Group condition are shown in dark blue, and Self trials are in light blue. The orange inset of each plot shows the average difference (Group minus Self) for the corresponding measurement of each trial. Error bars indicate the SEM across participants. (**A**) There is no significant difference in accuracy between the Group and Self conditions. (**B**) Participants have lower confidence when taking responsibility for others compared to when deciding for themselves alone, despite receiving explicit feedback that they perform equally well in both conditions. (**C**) In contrast to the previous experiments, differences in RTs between Group and Self trials in the feedback experiment did not reach traditional significance levels (main effect of condition: $\beta_{3.1}=0.0064$, 95% CI = [−0.0038, 0.0163], $P_{\text{MCMC}}=0.1$).

In the task with feedback, people’s confidence is enhanced compared to without feedback, but they still have a lower confidence with responsibility for others. We first compared the overall effect of the feedback treatment across tasks using Eqs. 4 and 5. Feedback did not significantly improve participants’ accuracy (main effect of feedback $\beta_{4.3}=0.04$, 95% CI = [−0.02, 0.09], $P_{\text{MCMC}}=0.09$) but did enhance participants’ confidence relative to when no feedback is given (main effect of feedback $\beta_{5.3}=0.10$, 95% CI = [0.10, 0.11], $P_{\text{MCMC}}<0.001$), which replicates the finding in (*30*). Next, we compared the Group and Self conditions within the feedback treatment group. Only 2 of 108 participants showed a difference (P<0.05) in accuracy when testing within each person separately, which means that the feedback worked as expected to show participants that their performance in two conditions was similar (Fig. 5A). Nevertheless, we found that participants still had lower confidence when responsible for others (Fig. 5B, main effect of condition $\beta_{2.1}=-0.06$, 95% CI = [−0.08, −0.03], $P_{\text{MCMC}}<0.001$). Furthermore, the effect of social responsibility does not decrease across trials (interaction effect of trial number and condition: $\beta_{8.4}=0.00015$, 95% CI = [−0.00035, 0.00073] $P_{\text{MCMC}}=0.24$). In other words, participants’ confidence reports do not change as they receive more evidence that their performance is similar in each condition. In addition, just as in the experiments without feedback, the participants’ change in confidence was uncorrelated with their change in accuracy (Spearman’s rank correlation: ρ=−0.103, P=0.291). These results imply that the decreased confidence with social responsibility cannot be rectified simply through feedback about performance.

Although participants remained underconfident when responsible for the entire group, performance feedback did lead to accurately calibrated confidence in Self trials. Using simulations, we found that participants’ behavior is consistent with unbiased (i.e., accurately calibrated) confidence in the Self condition in the feedback treatment (main effect of “simulation with fitted parameter in Self condition” compared to “simulation with no metacognitive bias” $\beta_{7.1}=-0.05$, 95% CI = [−0.15, 0.06], $P_{\text{MCMC}}=0.19$). This replicates the finding from (*29*) that feedback can reduce metacognitive bias. However, changes in metacognitive bias still lead to lower confidence under social responsibility even with feedback (fig. S3). Specifically, simulations using the combination of the fitted metacognitive bias parameters (additive and multiplicative) produce lower levels of confidence in the Group compared to Self trials (main effect of condition $\beta_{2.1}=-0.04$, 95% CI = [−0.08, 0.01], $P_{\text{MCMC}}=0.04$).

While performance feedback did not change the effect of social responsibility on confidence, it did reduce the effect on decision RTs (Fig. 5C). In the feedback experiment, the influence of the Group condition on prolonging RT is reduced (interaction effect of Group condition and experiment type: $\beta_{6.4}=-0.01$, 95% CI = [−0.0305, 0.0021], $P_{\text{MCMC}}=0.05$), although people still spent a longer time to initiate the stimulus display (main effect of condition $\beta_{3.1}=0.03$, 95% CI = [0.01, 0.05], $P_{\text{MCMC}}>0.001$). Meanwhile, the correlation between individual confidence change and log-transformed RT change is not significant (Spearman’s rank correlation ρ=−0.13, P=0.17) in the experiment with feedback. These results suggest that the lower confidence in the Group condition does not always stem from the same mechanism that determines decision RTs, and there may be a distinct effect of social responsibility on metacognitive bias.

### Individual perceptions of social responsibility

Participants that reported feeling more responsibility in the Group condition tended to have greater decreases in confidence on Group compared to Self trials. Following the experiment, participants completed a survey in which they indicated whether they felt more, the same, or less responsible in the Group condition. Across all versions of the task (*n* = 316), 241 participants reported that they felt more responsible in Group condition, 74 participants reported that they felt the same level of responsibility, and 1 participant reported feeling less responsibility in Group condition. People who recalled feeling subjectively the same levels of responsibility in the Group and Self conditions still had lower confidence in the Group condition (main effect of condition $\beta_{9.2}=-0.03$, 95% CI = [−0.06, −0.01], $P_{\text{MCMC}}=0.01$), although confidence tended to decrease more for the people who recalled feeling more responsibility in the Group condition (interaction effect $\beta_{9.4}=-0.02$, 95% CI = [−0.05, 0.01], $P_{\text{MCMC}}=0.11$). These results show that most participants perceive different degrees of responsibility when taking responsibility for others, and that perceptions of responsibility might affect confidence when making decisions that determine the benefits to other people.

We also examined the influence of social affiliation on the change in confidence. All participants went through the same group induction phase, but they still had different degrees of affiliation with the people whom their decisions would affect. We measured the degree of social affiliation through a self-reported affiliation score and behavior in a dictator game with in-group versus out-group members. We found that there was a significant correlation between the self-reported social affiliation score and the change in confidence under social responsibility in all three versions of the task. People who are more affiliated with the in-group members had a greater decrease in confidence when their decisions affected those group members (Spearman’s rank correlation in unlimited display task: ρ=−0.300, P=0.002; in limited display task: ρ=−0.188, P=0.014; in feedback task: ρ=−0.148, P=0.062). Within the dictator game, there was no significant correlation between the difference in the amount of money shared with in-group members and out-group members and confidence changes. This distinction between affiliation scores and dictator game behavior is consistent with previous reports indicating that self-report measures often explain individual variability better than performance in standard laboratory tasks (*31*, *32*).

### A normative model of delegation with confidence

Often potential decision-makers have the option to decide upon an action themselves or to delegate some or all of the decision-making process to others. We hypothesized that confidence will affect the decision to delegate to others to avoid responsibility. We constructed a normative model based on this hypothesis. Essentially, we conceptualize the process of resolving the decide-alone versus delegate trade-off as analogous to an information-seeking problem (see Materials and Methods for the mathematical expressions of the normative model). The option to delegate introduces a two-step decision-making process. Initially, participants make an evaluation of the options, forming a basis for their confidence in both their own potential decision and in potential delegates’ performance. Subsequently, they face a decision to either (i) “lead” by adhering to their own choice or (ii) delegate by adopting the delegates’ decision.

In addition, we posit that individuals may exhibit a preference to delegate decisions or to make decisions themselves. We will refer to this preference as “delegation preference.” Delegation preference could represent a cost and/or a benefit. For example, delegation may incur a subjective cost because the potential decision-maker relinquishes control, or delegation may offer a subjective benefit in that the individual is absolved from the responsibility for the outcome. In addition, the delegation preference quantified by the model can be interpreted as reflecting the optimality of delegation behavior with regard to decision accuracy, where neutral delegation preference ($\phi_{d}=0$) represents behavior that will yield the highest possible monetary reward, conditional on the confidence accurately reflecting the potential delegates’ and one’s own accuracy, as shown through empirical tests in the Supplementary Text.

**Fig. 6.**
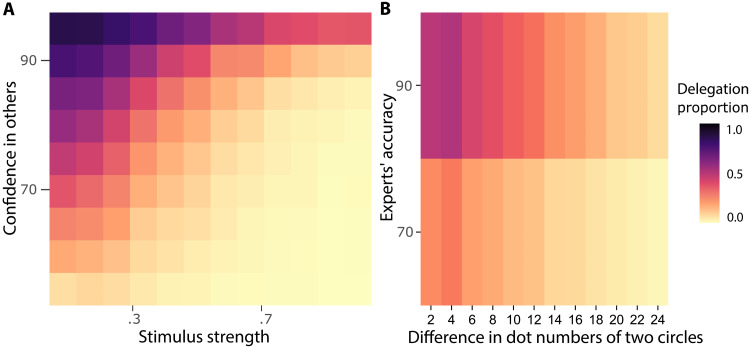
Delegation decisions depend on confidence in self and potential delegates. The heatmaps show a model simulation (**A**) and empirical data (**B**) of delegation proportion across stimulus strength (difference in dot numbers of two circles) and confidence in others (experts’ accuracy). The heatmap color represents the average delegation proportion in the corresponding category. (A) The *x* axis represents the stimulus strength normalized to range between 0 and 1, with larger values representing easier trials; the *y* axis represents confidence in others with 50 indicating complete lack of certainty and 100 indicating complete certainty. The simulation was done with the delegation preference being neutral. The heatmap values indicate the average delegation proportion across 1000 simulations per cell. (B) Participants’ delegation behavior corresponds closely with simulated behavior from our model shown in (A). The heatmap values in (B) represent the average delegation proportion across participants.

According to the normative model, individuals are more inclined to delegate decisions to others when they have lower confidence in their own decision, and/or they have higher confidence in those to whom they might delegate, and/or exhibit a stronger delegation preference toward delegation, as shown in the simulations based on our model (Fig. 6A). To test the ability of our model to explain human behavior, we examined behavior in a delegation task together with the limited–display-time version of the forced-choice task reported above. The differences between the delegation and forced-choice versions of the task is that the delegation task adds a third option to delegate to a panel of experts rather than making the decision oneself, and there are no confidence ratings after choices. In each trial, participants were informed that the current expert panel had an accuracy of 70 or 90% simultaneously with stimulus onset. The order of the delegation and forced-choice tasks was counterbalanced across participants.

First, we tested the basic prediction that delegation should be associated with decision difficulty or confidence, independent of social responsibility. Figure 6B shows that as the trials become easier, participants tend to delegate the decision to experts less (main effect of stimulus strength: $\beta_{10.2}=-0.96$, 95% CI = [−0.99, −0.93], $P_{\text{MCMC}}<0.001$). This trend can be seen in both Experts 70 and 90% trials. This trend corresponds closely with simulated behavior from our model (Fig. 6A). Furthermore, we used a Bayesian hierarchical instrumental variable (IV) regression to test for a causal effect of confidence on delegation (Eqs. 13 and 14; main effect of confidence for Experts 70% trials: β=−0.70, 95% CI = [−0.74, −0.67], $P_{\text{MCMC}}<0.001$; main effect of confidence for Experts 90% trials: β=−0.73, 95% CI = [−0.76, −0.70], $P_{\text{MCMC}}<0.001$). Consistent with this result, we also found that people who are generally more confident in the forced-choice task are less likely to delegate to the experts in the delegation task (Spearman’s rank correlation ρ=−0.262, P=0.004), which is also consistent with our model’s predictions.

**Fig. 7.**
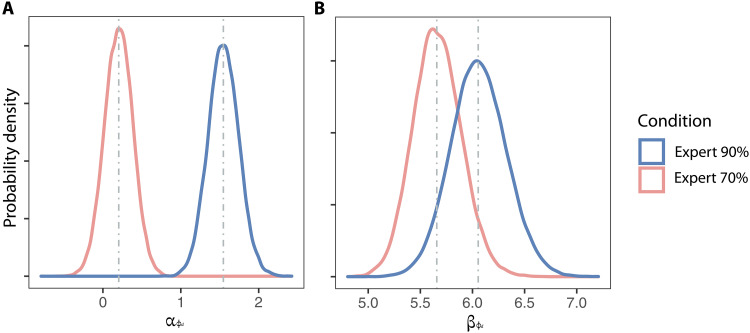
The constant delegation preference term is stronger when experts are more competent. The density plots show the fitted population-level posterior distributions of $\alpha_{\phi_{d}}$ and $\beta_{\phi_{d}}$ for each level of experts. Dotted gray lines represent the medians of fitted posterior chains. Different colors represent different expert levels. (**A**) $\alpha_{\phi_{d}}$, the constant delegation preference term in Experts 90% trials is higher than that for the Experts 70% trials. (**B**) $\beta_{\phi_{d}}$, the confidence-in-self–dependent coefficient in the Experts 90% trials is not significantly larger than that of the Experts 70% trials.

Next, we tested how delegation decisions are related to experts’ accuracy. The overall probability of delegating to Experts 70% was lower compared to Experts 90% (main effect of expert $\beta_{10.3}=-1.34$, 95% CI = [−1.41, −1.27], $P_{\text{MCMC}}<0.001$). In both Experts 90% and Experts 70% trials, participants’ decisions to delegate were based on the winning model of delegation preference, which includes both confidence-dependent and constant parameters (table S4; see Materials and Methods for the mathematical expressions for different constructions of delegation preference). We found that the strength of constant term for the delegation preference differed between the expert types. Figure 7 shows that the coefficient, $\alpha_{\phi_{d}}$, for the constant delegation preference term in Experts 90% trials is higher than that for the Experts 70% trials ($P_{\text{MCMC}}<0.001$), while the confidence-in-self-dependent coefficient, $\beta_{\phi_{d}}$, in the Experts 90% trials is not significantly larger than that of the Experts 70% trials ($P_{\text{MCMC}}=0.128$). This indicates that the delegation preference is dependent on the level of confidence in the people whom decision-makers could delegate to, and participants are more likely to opt for delegating when their confidence in the delegate’s performance is higher.

### Delegation levels increase with lower confidence under social responsibility

**Fig. 8.**
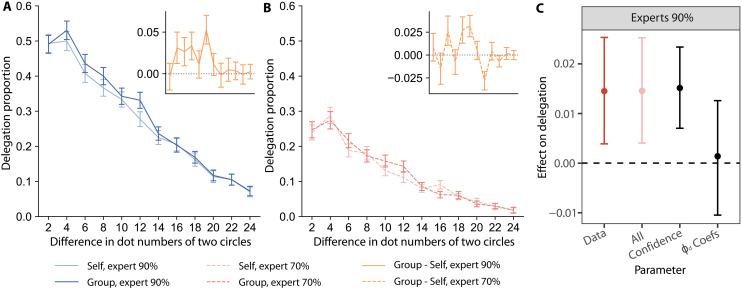
The delegation model explains the observed patterns of delegation behavior. Delegation proportions decrease when decisions are easier (greater dot difference) and increase in Group compared to Self trials with 90%-accuracy experts. (**A**) The *y* axis shows the mean delegation proportion across participants during Expert 90% trials as a function of the difference in dots between the two stimuli along the *x* axis. The orange inset shows the average difference (Group minus Self) in delegation proportion of each trial. (**B**) The same as (A) but for Expert 70% trials. Delegation proportions are lower for 70 than 90% experts. (**C**) The error bar plots show the result of parameter sensitivity analyses testing of which the inputs or parameters in the delegation model are required to reproduce the empirical effects. The effect of interest on the *y* axis is the influence of the Group condition on delegation proportion, estimated from the generalized linear mixed model in Eq. 11. The *x* axis shows the effect in the empirical data, data, and simulations from the delegation model under different specifications. The simulations are labeled as follows: All includes changes in both confidence (input) and delegation preference coefficients (fitted parameters), “Confidence” includes differences in the measured confidence in the forced-choice task for corresponding conditions while leaving all free parameters fixed across conditions, “$\phi_{d}$ Coefs” keeps confidence fixed across conditions and uses delegation preference coefficients ($\alpha_{\phi_{d}}$ and $\beta_{\phi_{d}}$) fit separately to each condition. Error bars represent the 95% CI of the effect on delegation.

As shown in Fig. 8A, in the delegation task, people delegated more to experts with 90% accuracy in the Group compared to the Self condition (main effect of condition $\beta_{11.1}=0.12$, 95% CI = [0.04, 0.20], $P_{\text{MCMC}}<0.001$). This increase in the delegation proportion when responsible for others replicates the findings of responsibility aversion in previous work on risky choices (*9*). In contrast to the Experts 90% trials, there was no difference in the delegation proportion in Experts 70% trials across the two responsibility conditions (Fig. 8B; main effect of condition $\beta_{11.1}=0.03$, 95% CI = [−0.11, 0.17], $P_{\text{MCMC}}=0.36$). However, there is a positive correlation between an individual’s change in delegation proportion for experts with 90% accuracy and experts with 70% accuracy (Spearman’s rank correlation ρ=0.240, P=0.009), suggesting a common mechanism underlying responsibility aversion in both expert performance levels.

According to the normative delegation model, there are two possible drivers of responsibility aversion: (i) lower confidence when taking responsibility for others and (ii) changes in the constant or confidence-in-self–dependent coefficient terms for the delegation preference ($\alpha_{\phi_{d}}$ or $\beta_{\phi_{d}}$) when taking responsibility for others. To test whether the change in confidence between conditions is related to responsibility aversion, we computed a Bayesian mixed effect model (Eq. 12) and found that lower confidence was a significant predictor of more delegation in the Experts 90% condition (main effect of confidence: $\beta_{12.1}=0.11$, 95% CI= [0.02, 0.20], $P_{\text{MCMC}}=0.01$). In contrast, the change in confidence is not a significant predictor in the Experts 70% condition (main effect of confidence: $\beta_{12.1}=0.02$, 95% CI = [−0.05, 0.09], $P_{\text{MCMC}}=0.27$). Using the parameter sensitivity analysis method for the normative delegation model (Fig. 8C), we can see that the change in confidence alone can explain the higher delegation proportion in Group condition with Expert 90%, even when holding delegation preferences constant across conditions.

### Nonzero delegation preferences lead to less accurate decision performance

A participant’s delegation preference quantified by the model can be interpreted as reflecting the optimality of delegation behavior with regard to decision accuracy. If a person’s confidence in their own decision-making accurately represents the true accuracy of their decision, opting to delegate to experts when the experts’ accuracy is greater than the confidence in oneself can be viewed as selecting the action that maximizes the decision accuracy. Consequently, a neutral delegation preference ($\phi_{d}=0$) represents behavior that will yield the highest possible reward, conditional on an accurate calibration of confidence.

Our data show a negative association between delegation preference and final accuracy (the total accuracy of both delegated and led decisions). Note that this relationship holds despite participants’ biases in metacognition (i.e., miscalibration of confidence). As predicted by the model, we found that there is negative correlation between participants’ absolute delegation preference and their final accuracy (fig. S4A; Expert 70%, Group condition: Spearman’s rank correlation ρ=−0.224, P=0.007; Expert 70%, Self condition: ρ=−0.263, P=0.002; Expert 90%, Group condition: ρ=−0.260, P=0.002; Expert 90%, Self condition: ρ=−0.241, P=0.004). Here, the delegation preference $\phi_{d}$ is derived from the fitted $\alpha_{\phi_{d}}$, $\beta_{\phi_{d}}$, and participants’ reported confidence. For the Experts 70% trials, we restricted our analysis to difficult trials (where the difference in the number of dots between the two circles was less than or equal to 8). This is because, in easy trials, participants’ confidence levels were substantially above 70, and thus the magnitude of $\phi_{d}$ would have little influence on their delegation decisions (as shown below, most participants exhibit a bias towards leading, with $\phi_{d}$ being smaller than 0). For the Experts 90% condition, we use all the trials because participants’ average confidence is often near or below 90 even when the dot difference is large. Lastly, to test the robustness of our results, we computed the out-of-sample performance of a simple prediction model that used the correlation between absolute delegation preference and final accuracy in leave-one-out fashion (see Materials and Methods for more details). The correlation between out-of-sample predictions and true final accuracy was significant in each of the four responsibility × expert-level task conditions (Expert 70%, Group condition: Pearson correlation ρ=0.183, P=0.024; Expert 70%, Self condition: ρ=0.301, P<0.001; Expert 90%, Group condition: ρ=0.292, P<0.001; Expert 90%, Self condition: ρ=0.390, P<0.001).

### Do social responsibility effects simply reflect the amount of reward at stake?

The total potential payoff across all group members is higher in the Group than Self conditions of the forced-choice and delegation tasks. Thus, it is possible that the apparent effects of social responsibility on decision and metacognitive processes are due (in part) to the higher monetary stakes present in the Group trials. To test this hypothesis, we designed a nonsocial version of our task in which people make decisions with either 15- or a 60-point stakes (700-ms stimulus display time, no feedback on choice accuracy). These stakes mirror the total potential payoffs across all people in the Self (15 points × 1 participant) and Group (15 points × 4 participants) conditions of the social responsibility experiments.

**Fig. 9.**
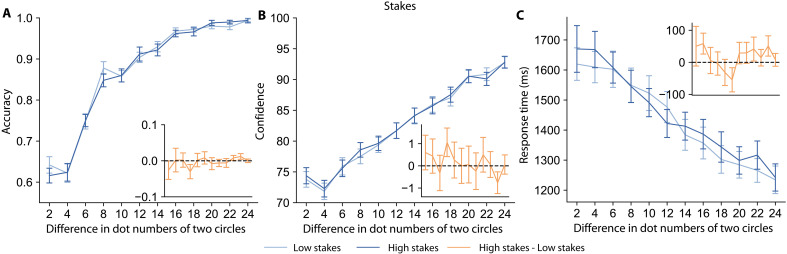
Monetary stakes alone do not change confidence. The plots show accuracy, confidence, and RTs across various stimulus strengths (difference in dot numbers of two circles) in High-stake and Low-stake conditions for the stakes task. High-stakes trials are shown in dark blue, and Low-stakes trials are in light blue. The orange inset of each plot shows the average difference (high minus low) for the corresponding measurement of each trial. Error bars indicate the SEM across participants. (**A**) There is no significant difference in accuracy between the High-stake and Low-stake conditions. (**B**) There is no significant difference in confidence between the High-stake and Low-stake conditions. (**C**) Participants spent a longer time responding when the stakes are higher.

As shown in the Fig. 9, we found that neither accuracy (main effect of condition $\beta_{1.1}=-0.04$, 95% CI = [−0.15, 0.08], $P_{\text{MCMC}}=0.27$) nor confidence (main effect of condition $\beta_{2.1}=0.01$, 95% CI = [−0.03, 0.05], $P_{\text{MCMC}}=0.69$) is significantly different between the high and low stake conditions. However, people took longer to make their decisions (main effect of condition $\beta_{3.1}=0.01$, 95% CI = [0.0019, 0.0276], $P_{\text{MCMC}}=0.01$) and to initiate the stimulus display (main effect of condition $\beta_{S2.1}=0.04$, 95% CI = [0.01, 0.07], $P_{\text{MCMC}}=0.01$) in high-stakes trials. These results indicate that having responsibility for others shares certain features with the stakes component of social responsibility, particularly in that individuals spend more time making decisions and preparing themselves before starting the trials in both cases. However, the impact on confidence appears to be unique to situations where individuals are responsible for the welfare of others. These findings echo previous literature, which shows that decisions affecting others differ from decisions affecting only the decision-maker, even when controlling for payoff magnitude (*3*).

## DISCUSSION

Our study demonstrates and explains changes in decision behavior when individuals take responsibility for others. Specifically, social responsibility causes a reduction in confidence by changing decision-makers’ metacognitive bias. These metacognitive changes occur in basic magnitude judgments that do not entail risk or loss. Thus, our work shows that responsibility affects fundamental decision mechanisms beyond revealed risk or loss preferences.

Previous studies of responsibility and decision-making have focused almost exclusively on its influence on risk preferences and loss aversion. Those studies have yielded mixed and sometimes conflicting results. Responsibility for others can take various forms that may determine, in part, how it influences the decision process. The effects of responsibility on decision-making differ as a function of payoff inequality (whether and to what extent the representative’s decision affects only the recipient or also the representative) (*3*), as well as in the domains of loss/gain and reciprocity (*33*). Here, we have focused on scenarios that isolate effects of responsibility itself by ensuring that the decision-maker and the affected group share the same consequences of the decisions. Although even in this restricted domain, the results of social responsibility on risk preferences are mixed, they typically indicate that people are more risk or loss averse when making decisions that affect others (*3*–*8*).

Changes in confidence may explain some of the previous findings on risky choices and social responsibility. The previous studies on responsibility and risk did not collect data on participants’ decision confidence. However, a separate line of work has shown that choosing a risky over a safe option is associated with lower confidence (*13*). Together with our results showing that social responsibility reduces confidence in nonrisky decisions, the available data suggest the intriguing hypothesis that people prefer safe over risky options when responsible for others to avoid a “double hit” to their confidence. There is a positive correlation between confidence and affect (*34*): Feeling more confident is frequently described as rewarding (*35*), whereas low confidence is associated with increased negative affect and heightened anxiety (*36*). Therefore, it is conceivable that past findings indicating that increases in risk aversion under social responsibility are not driven (entirely) by attitudes toward risk but rather reflect the goal of maintaining confidence. Social responsibility may have distinct effects on both risk and confidence; however, the parsimonious possibility that its influence on confidence alone could explain the effects observed across risky and nonrisky choices merits further investigation.

The effect of responsibility on confidence may work through channels related to intrinsic and/or extrinsic others-concern motivations. Extrinsic others-concern motivations include aspects such as the possibility that decision-makers may anticipate being blamed by others when their decisions are suboptimal. Examples of intrinsic others-concern motivations include decision-makers experiencing guilt or self-blame for causing losses to others, even in the absence of explicit social pressure (*3*). Wang and colleagues (*3*) showed that both types of motivations can lead group representatives to exhibit greater risk aversion. These findings may explain the reduction in confidence seen in our task, as individuals might feel uncertain about whether their performance aligns with the “performance standard” of the group.

Furthermore, beyond the average effect of responsibility, we found that people who are more affiliated with their experimentally assigned group members following the group-induction phase had a greater decrease in confidence when their decisions affected their group members. These results are consistent with a large literature showing that decision-making and confidence are strongly influenced by both individual and contextual factors. For example, people tend to be more confident in their choices when aiming to achieve gains than when trying to avoid losses, despite equivalent levels of objective difficulty and learning performance (*37*–*39*). Social contexts also play a substantial role in influencing decision-making (*40*–*45*) and metacognition (*46*–*48*). Fareri and colleagues (*4*) showed that people are more risk-neutral and consistent when making choices for friends as opposed to strangers. Our findings are consistent with this work in showing that social identity and affiliation modulate the effect of responsibility for others.

Behavior may be affected by confidence at multiple levels. In this study, we focus on local decision confidence, which is elicited at or around the time of a specific decision. In addition, global self-performance estimates (SPEs), global beliefs, or self-efficacy may play a role in decision-making under responsibility as well. Although global SPEs and local decision confidence are argued to have at least partially distinct mechanisms (*49*, *50*), the two appear to have a bidirectional influence on each other. Global beliefs about one’s abilities can affect effort and task performance (*51*–*53*), which would affect local confidence. On the other hand, local decision confidence has been shown to shape global SPEs (*30*) and share partially overlapping brain response patterns with global SPEs (*49*). Global SPEs are closely related to the concept of prior beliefs, and manipulating prior beliefs has been shown to causally induce under or overconfidence while maintaining similar performance accuracy (*54*). The version of our experiment that included feedback aimed to manipulate people’s beliefs by making them aware that their performance was consistent across different conditions. Despite this intervention, the confidence gap between Group and Self trials persisted. This finding indicates that global SPEs are unlikely to be the primary driver of responsibility effects. Nevertheless, it is possible that prior beliefs may play an important role in modulating the influence social responsibility has on local decision confidence.

The relationship we found between lower confidence and more delegation is in line with the established role of confidence in information seeking and control. Previous research has shown that confidence plays a role in multistage decision-making (*55*), with individuals tending to seek more information when they feel more uncertain about their choices (*14*–*17*), and various normative frameworks suggest that uncertainty is a critical factor in determining when and where information should be sought (*56*–*58*). We show that changes in confidence are associated with information seeking—delegating decisions to experts with known accuracy—cross task settings (Self trials versus Group trials). However, despite the similarities, there is an important difference between delegation in our experiments and typical information seeking tasks. Delegation involves complete transfer of control and elimination of the sense of agency (*59*). In contrast, typical information seeking tasks allow individuals to seek more information in nonsocial or social contexts (advice-seeking) while retaining control of the actions (*14*, *16*, *17*). Although social responsibility led to more delegation, participants in our tasks demonstrated a clear preference to maintain control (i.e., had negative delegation preference, on average) both with and without responsibility for others. This reluctance to yield decision power to the experts resulted in less success, and consequently, less monetary reward in the delegation task.

Differences in decision RT between conditions with and without social responsibility might be explained through the framework of resource rationality (*60*). Resource rationality posits that decision-makers aim to maximize the difference between the value of choosing correctly and the cost of acquiring the necessary information to do so. In the Group condition of the social experiments, participants who feel a greater sense of responsibility may treat the potential payoff for being correct as if it were higher compared to the Self condition. This is because the payoff across all group members in the Group condition is equal to the payoff for oneself plus the payoffs to other group members, although subjectively others’ payoffs may potentially be discounted relative to one’s own. Correspondingly, people may allocate more cognitive resources to performing the task in the Group condition, as evidenced by longer RTs. Our nonsocial control task manipulated the monetary value of making the correct choice. Consistent with resource-rational predictions, participants in those monetary stakes experiments took more time to respond—i.e., allocated more resources to acquiring information—in the high versus low payoff trials. Critically, however, this additional decision time did not translate into better performance or higher confidence in either the social responsibility or monetary stakes tasks. Pure monetary stakes did not influence confidence, and confidence was lower despite longer RTs in the social responsibility trials. In that sense, it was not a beneficial allocation of resources in either context.

However, it may have been difficult for participants to access the return on their resource investments in the tasks without performance feedback. Notably, the difference in decision RTs between Group and Self trials was significantly reduced when we added performance feedback to the social responsibility task. The feedback may have allowed participants to learn that allocating additional time (i.e., resources) to making the magnitude judgments did not lead to better performance, and thus they did not do it. A resource-rational explanation for RT differences that is distinct from the effect of responsibility on confidence could explain why the two effects coincided in versions of the task without feedback, but only confidence differed between Group and Self trials when participants received feedback on their accuracy.

In summary, we have shown that responsibility for others leads to a decrease in decision confidence irrespective of behavioral performance. This decrease in confidence is the primary cause of responsibility aversion. Although overall confidence is improved when people are given more time to make decisions or feedback on their performance, confidence still remains lower when decision-makers are responsible for others compared to themselves alone. Our results show that the effects of responsibility extend beyond the field’s previous focus on risk and loss preferences and raise the intriguing possibility that apparent changes in such preferences are driven, at least in part, by underlying changes in confidence. Real-world decision-making often involves planning, reasoning, and integration or trade-offs between multiple objectives, and it is likely that some aspects of more complex decision problems may modulate the effects of social responsibility on confidence. However, the capacity to reflect on, evaluate, and control mental function is present across the spectrum of decision complexity. Thus, understanding how social responsibility shapes confidence offers the potential for a unifying framework for interpreting decision-making under responsibility across diverse decision-making contexts.

## MATERIALS AND METHODS

### Experiment with limited display time

#### Participants

There were two waves of data collection for this version of the task for replication purposes; the statistical results for the effect of condition hold in both waves, and in Results, we report the results of analyzing both waves together for clarity and conciseness. In wave 1, a total of 40 participants took part in the experiment. In wave 2, a total of 100 participants took part in the experiment; of these, we excluded 5 participants because of their inability to understand the experiment, as indicated by their responses in the experiment feedback survey and task comprehension quiz. Moreover, because of technical issues resulting in data saving failures, we were compelled to discard the data from the delegation task for 21 participants who took part in the same experiment session. As a result, we included data from 95 participants in the forced-choice task (i.e., without a delegation option) and data from 77 participants in the delegation task from wave 2. All participants provided their written informed consent in accordance with the procedures established by the Institutional Review Board of the Faculty of Business, Economics, and Informatics at the University of Zurich and the Ethics Committee of the Canton of Zurich (protocol number: OEC IRB # 2020-061).

#### Procedure

The whole experiment involved a series of tasks. The main task was the magnitude judgment experiment.

*Group induction*. To create a sense of cohesion among groups of four strangers, the experiment began with a group induction procedure. The procedure followed standard group induction protocols (*61*). Each group was seated together and assigned a colored ID tag identifying their group. Participants were first instructed to give a 2-min introduction about themselves within each group. The groups were then informed that they would complete two games as a team and that their group’s performance would be compared to other groups in this session. The group with the highest score would earn a bonus of 28 CHF. The first game was called “same but different,” and required the group to complete a form in 8 min listing things that all group members had in common but also unique for each of them; for example, they could all study at the same university but have different majors. The more entries they filled in, the more points the group would earn. The second game was a 10-min quiz containing 20 general questions about music, art, history, and science that the group members completed together. To avoid influencing the outcome of the experiment, the identity of the winning group was not revealed to participants until the end of the experiment. This type of group induction procedure is commonly used (*61*) to establish a minimal level of acquaintance among ex-ante strangers. After completing the group induction phase, each participant was seated in a separate cubicle and performed the following tasks independently.

##### Forced-choice magnitude judgment task

1) Stimuli. The stimuli consisted of two circles with dots in each circle. Participants were required to decide which of the two circles contain more dots. One of the two circles always contained 50 dots while the other contained 50 + *c* dots, where *c* falls between 2 and 24 with a step of 2, resulting in 12 difficulty levels. All dots were of the same size, and their locations were randomly generated using a uniform distribution on a circular space. The position of the circle containing the higher number of dots was randomly assigned to be on the left or right on each trial. The order of the trials of varying difficulty was randomized for each participant.

2) Confidence elicitation. After the participants made their decision, they were asked to rate their confidence in their decision with an incentivization mechanism, which was designed to encourage them to truthfully reveal their subjective probability of success. This was done using the matching probability rule, as described by (*22*). Under this rule, participants’ payment depends on the accuracy of their stated confidence. We chose this confidence elicitation method because it has been shown to be the most effective mechanism for eliciting beliefs, demonstrating strong performance in calibration and discrimination, consistent elicitation across different measures and tasks, and solid empirical and theoretical foundations (*23*). Critically, this confidence elicitation procedure has also been shown to be robust to the participants’ risk preferences both theoretically and empirically (*21*–*24*).

The matching probability rule operated as follows: A random number $n_{1}$ between 1 and 100 was generated for each trial. If the participant’s reported confidence (probability of success, *p*) was greater than $n_{1}$, the computer checked to see whether their choice of the circle was correct. If it was, they won 15 CHF; if it was not, they got 0 CHF. If *p* was less than $n_{1}$, a second random number $n_{2}$ was drawn. If $n_{2}$ was less than or equal to $n_{1}$, the participant won 15 CHF, otherwise they got 0 CHF. This rule can be understood as follows: The higher the initial rating of *p*, the more likely the correctness of the decision will determine earnings. The lower the rating, the more likely earnings will be determined by chance (the second number). A particular rating value (e.g., 70%) thus reveals how participants trade off a belief in their decision being correct against a randomly determined reward. Note that this mechanism is a proper scoring rule and provides incentives for a subject to reveal true beliefs regardless of their preferences. Specifically, the expected reward for this mechanism with a subjective rating *p* and a probability of success *s* is $p\times \left[15\times s+0\times \left(1-s\right)\right]+\left(1-p\right)\times \left[15\times \frac{1+p}{2}+0\times \frac{1-p}{2}\right]$, which achieves its maximum for p=s. Before the experiment, we had explained the various possible outcomes of this confidence elicitation mechanism to participants, along with their intuitive interpretation so that they understood how different ratings would affect their potential earnings, how over- or underreporting confidence would lead to nonoptimal payoffs, and why it is in their financial interests to report their true beliefs.

3) Each trial consisted of the following procedure: First, two outline circles (each with a diameter of 7.7 cm) were displayed on the screen, with fixation crosses at their centers, and a horizontal distance of 8.55 cm between them. The participants were positioned approximately 55 cm away from the monitor. They were instructed to find a comfortable seating position to ensure that they could observe both circles without shifting their gaze. The participants were free to initiate the trial by pressing the “Space” key on a standard computer keyboard when they were ready. Small dot stimuli (diameter 0.2 cm) then appeared inside each circle for 700 ms. The participants were asked to indicate which circle, left or right, contained a higher number of dots by pressing the “j” or “l” keys, respectively. They had no time limit to respond. After responding, they were asked to indicate their level of confidence in their choice (from 50 to 100% in steps of 10%) using the 1 to 6 number keys, again with no time limit on the response. No feedback was given following either choices or confidence ratings.

4) This task included two conditions: the Self condition and the Group condition. In the Self condition, the participant’s action only affected their own monetary payoff. In contrast, in the Group condition, the outcome of the action affected not only the target participant but also their group members. For example, if a participant obtained a reward of 15 CHF, that same amount would be applied as the payoff of each of the four group members for this condition. Apart from this, the matched Group and Self trials were identical in every other aspect. During the task, there was no interaction between the individuals, and participants could not influence the other group members’ decisions.

The participants were informed that at the end of the whole experiment, the amount of reward they earned in Group trials would be announced in front of their corresponding group. In the payment stage, one participant of each group would be randomly selected to determine the final payment of all group members. The experimenter also announced which one of the group members was chosen randomly to determine everyone’s payment.

5) In this task, there were 10 trials for each difficulty level, for a total of 120 trials in each condition (Group or Self). The Group and Self trials were presented in blocks of 10 trials that were pseudo-randomly intermixed for each participant such that no more than three consecutive blocks were of the same condition. We informed the participants before the task that if they saw four animal icons on the left side of the screen, it indicated the “Group condition,” whereas if there was one animal icon on the left side, it indicated the “Self condition” (all icons in the experiment are designed by OpenMoji—the open-source emoji and icon project. License: CC BY-SA 4.0). We also informed them of the condition for the next 10 trials during each transition phase between blocks. For each condition, 10 trials were randomly selected and their averages were computed for payment calculation.

*Delegation task*. In this task, in addition to the option to choose the left or right circle, the participants also had the option to delegate the decision to a group of experts. There were two groups of experts that were randomly assigned to a trial: Experts I had an accuracy of 90%, which meant that their chance of being correct on a single trial was 90%. Experts II had an accuracy of 70%, which meant that their chance of being correct on a single trial was 70%. If a participant chose to delegate the decisions to experts, the reward would be determined by the correctness of the group of experts’ decision. The stimuli were shown for 700 ms, while participants were given unlimited time to make decisions on each of the trials and did not receive any feedback on the outcome of their choices. The Delegation task consisted of the same 120 trials from the baseline task repeated under both conditions and both expert levels, resulting in a total of 480 trials.

To fully alleviate the burden of responsibility for others with delegation, only the joint accuracy (across both conditions) for trials where each group member made decisions independently (i.e., did not delegate to experts) was announced during the final payment stage after the completion of the study. This design was used to allay any concerns that the decision to delegate still resided under the participants’ responsibility and to simulate real-life situations where others typically remain unaware of your performance if you do not assume the responsibility-taking role. The randomly chosen participant determining the payment for the whole group was also announced. Trials in which participants passed the decision to experts were not factored into each participant’s announced accuracy but still affected the payment for themselves or the whole group, depending on the condition.

The order in which the participants completed the forced-choice task and the delegation task was counterbalanced. Half of the participants completed the forced-choice task first, followed by the delegation task, while the other half completed the delegation task first, followed by the forced-choice task.

*Social preference measures*. After completing the delegation task, the participants performed an anonymous dictator game task, in which they received an endowment of 7 CHF and were asked to allocate a portion of this money to a randomly selected member of their group. This procedure was then repeated with a randomly selected member of an out-group from the same experimental session. In addition, the participants were asked to rate their feelings of affiliation with their in-group and out-groups on a 1 to 10 scale.

*Feedback survey*. At the end of the experiment, the participants filled out a feedback survey in which they were queried on how they made decisions and rated their confidence, as well as their sense of responsibility toward others.

### Experiment with unlimited display time

#### Participants

There were two waves of data collection for this version of the task for replication purposes; the statistical results for the effect of condition hold in both waves, and in Results, the data from wave 2 were shown for better visualization purposes (detailed explanation in the task procedure below). In wave 1, a total of 48 participants took part in the experiment, and of these, we excluded 2 participants because of their inability to understand the experiment, and another three participants because they were trying to solve the task by counting the dots and were extremely slow. In wave 2, a total of 52 participants took part in the experiment; of these, we excluded 1 participant because of their inability to understand the experiment. All participants provided their written informed consent in accordance with the procedures established by the Institutional Review Board of the Faculty of Business, Economics, and Informatics at the University of Zurich and the Ethics Committee of the Canton of Zurich (protocol number: OEC IRB # 2020-061).

#### Procedure

The series of tasks follow the same procedure as the experiment with limited display time, with two exceptions: (i) The stimuli were shown for an unlimited time until participants respond, instead of being shown for only 700 ms; (ii) there was no delegation task. In addition, there was a difference in the task setting between the two waves of data collection: In wave 1, only trials corresponding to the easier half-range of the difficulty were shown to save experiment time, and there was no indication to encourage participants to make decisions within a reasonable time. In wave 2, trials corresponding to the full difficulty range were used, and participants were shown a warning if they took longer than 20,000 ms (around 3.33 min) to make a decision.

### Experiment with feedback

#### Participants

There were two waves of data collection for this version of the task for replication purposes; the statistical results for the effect of condition hold in both waves, and in Results, we report the results of analyzing both waves together for clarity and conciseness. In wave 1, a total of 60 participants took part in the experiment, and of these, we excluded 2 participants because of their inability to understand the experiment. In wave 2, a total of 52 participants took part in the experiment; of these, we excluded 2 participants because of their inability to understand the experiment. All participants provided their written informed consent in accordance with the procedures established by the Institutional Review Board of the Faculty of Business, Economics, and Informatics at the University of Zurich and the Ethics Committee of the Canton of Zurich (protocol number: OEC IRB # 2020-061).

#### Procedure

The series of tasks follow the same procedure as the experiment with limited display time, with two exceptions: (i) There is feedback on their accuracy both after they made choice and rated their confidence on each trial, and as a summary of each condition after every 10 trials. (ii) There is no delegation task.

### Experiment with different stakes

#### Participants

Fifty participants took this experiment. All participants provided their written informed consent in accordance with the procedures established by the Institutional Review Board of the Faculty of Business, Economics, and Informatics at the University of Zurich and the Ethics Committee of the Canton of Zurich (protocol number: OEC IRB # 2020-061).

#### Procedure

In this experiment, the participants did the task with forced choice and experiment feedback survey. In the task with forced choice, there are two conditions with either 15- or a 60-point stakes. These stakes mirror the reward structure in the Self (15 points × 1 participant) and Group (15 points × 4 participants) conditions of the social responsibility experiments. The stimulus was shown with 700 ms. No feedback was given following either choices or confidence ratings.

### Regression analyses

We used Bayesian hierarchical (generalized) regressions implemented with the brms package (*62*) in the statistical computing software R (*63*). For each model, we used three chains with 2000 samples per chain after burn-in. The $P_{\text{MCMC}}$ values reported for these regressions represent one minus the probability of the reported effect being greater (less) than zero given the posterior distributions of the fitted model parameters. We used an N(0,1) prior for all intercept terms (α) and slope coefficients (β), with the exception of the slope for difficulty, which was N(0.5,1).

#### The effect of stimulus strength and Group/Self condition on accuracy, confidence, and decision RT

We tested how stimulus strength (difference in the number of dots) and Group/Self condition were related choice to accuracy. The population-level regressors are listed in the equation below

$$\text{Correct}=\alpha_{1}+\beta_{1.1}\;\text{Condition}+\beta_{1.2}\;\text{Difficulty}+e$$ (1)

In this equation, *Correct* is a binary indicator of the choice accuracy. *Condition* is a binary variable indicating the Self and Group condition. *Difficulty* represents the standardized difference in the number of dots between two circles (across participants), unless noted otherwise. Subject-specific coefficients were estimated for all regressors except difficulty.

Similarly, we tested how stimulus strength and Group/Self condition were related choice confidence and decision RT. The population-level regressors are listed in the equations below

$$\text{Confidence}=\alpha_{1}+\beta_{2.1}\text{Condition}+\beta_{2.2}\text{Difficulty}+e$$ (2)

$$\text{logRT}=\alpha_{3}+\beta_{3.1}\text{Condition}+\beta_{3.2}\text{Difficulty}+e$$ (3)

In all equations, *confidence* is the standardized confidence rating in each trial, unless noted otherwise. *LogRT* is log-transformed decision RT in each trial.

#### Differences in accuracy, confidence, and decision RTs of each task

We tested how the participants’ choice accuracy, confidence, and decision RTs differ between tasks (e.g., limited versus unlimited display time, or with versus without feedback). The regressors are listed in the equations below

$$\begin{array}{cc} \text{Correct}= & \alpha_{1}+\beta_{4.1}\text{Condition}+\beta_{4.2}\text{Difficulty}\\ & +\beta_{4.3}\text{Task}+\beta_{4.4}\text{Task}\times \text{Condition}+e \end{array}$$ (4)

$$\begin{array}{cc} \text{Confidence}= & \alpha_{5}+\beta_{5.1}\text{Condition}+\beta_{5.2}\text{Difficulty}\\ & +\beta_{5.3}\text{Task}+\beta_{5.4}\text{Task}\times \text{Condition}+e \end{array}$$ (5)

$$\begin{array}{cc} \text{logRT}= & \alpha_{6}+\beta_{6.1}\text{Condition}+\beta_{6.2}\text{Difficulty}\\ & +\beta_{6.3}\text{Task}+\beta_{6.4}\text{Task}\times \text{Condition}+e \end{array}$$ (6)

In these equations, *Task* is a categorical variable that indicates the version of the task.

#### Testing the degree of underconfidence in Self trials

We tested the degree of underconfidence in Self trials in different versions of the experiment. To do this, we simulated confidence with no metacognitive bias and compared these values to simulations of confidence that used the fitted metacognitive bias derived from Self trials.

The regressors are listed in the equation below

$$\text{Confidence}=\alpha_{7}+\beta_{7.1}\text{SimType}+\beta_{7.2}\text{Difficulty}+e$$ (7)

In this equation, *SimType* is a categorical variable indicating the simulation type.

#### The effect of task progress on confidence in the task with feedback

We tested whether the Group/Self condition effect changed over the course of the experiment as participants experienced more trials and feedback. The population-level regressors are listed in the equation below

$$\begin{array}{cc} \text{Confidence}= & \alpha_{8}+\beta_{8.1}\text{Condition}+\beta_{8.2}\text{Difficulty}\\ & +\beta_{8.3}\text{trialInd}+\beta_{8.4}\text{trialInd}\times \text{Condition}+e \end{array}$$ (8)

Here, *trialInd* is the trial number indicating the progress of the task.

#### The effect of Group/Self condition and feeling of responsibility on confidence

We tested how the recalled feeling of responsibility related to the Group/Self condition influence on confidence. The population-level regressors are listed in the equation below

$$\begin{array}{cc} \text{Confidence}= & \alpha_{9}+\beta_{9.1}\;\text{Difficulty}+\beta_{9.2}\;\text{Condition}\\ & +\beta_{9.3}\text{Resp}+\beta_{9.4}\text{Resp}\times \text{Condition}+e \end{array}$$ (9)

In this equation, *Resp* is a binary indicator of whether people recalled feeling more (1) or same (0) level of responsibility in the Group compared to Self condition (We omitted the single participant who reported feeling less responsibility in Group trials from this analysis).

#### The effect of stimulus strength and expert levels on delegation

We tested how stimulus strength and expert levels were related to delegation. The population-level regressors are listed in the equation below

$$\begin{array}{cc} \text{Delegation}= & \alpha_{10}+\beta_{10.1}\text{Condition}+\beta_{10.2}\text{Difficulty}\\ & +\beta_{10.3}\text{Expert}+\beta_{10.4}\text{Expert}\times \text{Condition}+e \end{array}$$ (10)

In this equation, *Delegation* is a binary indicator for the choice in delegation task. *Expert* is a binary variable indicating the expert levels.

#### The effect of Self/Group condition on delegation

We tested how Self/Group condition was related to delegation by estimating the following regression separately for trials from each expert level. The population-level regressors are listed in the equation below

$$\text{Delegation}=\alpha_{11}+\beta_{11.1}\text{Condition}+\beta_{11.2}\text{Difficulty}+e$$ (11)

#### The effect of confidence change between conditions on responsibility aversion

The population-level regressors are listed in the equation below

$$\begin{array}{cc} \text{DelegationChange}= & \alpha_{12}+\beta_{12.1}\text{ConfidenceChange}\\ & +\beta_{12.2}\text{Difficulty}+e \end{array}$$ (12)

Here, *DelegationChange* represents the change in delegation proportion between Self/Group condition for each difficulty level, *ConfidenceChange* represents the change in standardized confidence ratings between Self/Group condition for each difficulty level. This model was fit independently for two accuracy levels of experts.

### IV regression to estimate the effect of confidence on delegation

To test whether subjective confidence exerted a causal effect on delegation, we used a hierarchical Bayesian IV model. Specifically, we used stimulus strength and Self/Group condition as exogenous instruments that we have shown to influence confidence and, for the purpose of the IV model, are assumed to affect delegation only through confidence. The IV model simultaneously estimates trial-wise confidence levels based on the difficulty and condition and tests the influence of this estimated confidence on the probability of deciding to delegate. Following our approach in the delegation model, we fit this IV regression model separately for each level of expert accuracy.

We observe data from i=1,…,N trials and j=1,…,J subjects. For each observation *i*, we have or generate

$$\begin{array}{cc} y_{i}\in \left\{0,1\right\} & :\text{binary outcome}\;\left(\text{delegate}\right)\\ c_{i}\in \mathbb{R} & :\text{continuous endogenous predictor}\;\left(\text{confidence}\right)\\ z_{i1}\in \mathbb{R} & :\text{instrument}\;\left(\text{difficulty}\right)\\ z_{i2}\in \left\{0,1\right\} & :\text{instrument}\;\left(\text{condition}\right)\\ j\left[i\right] & :\text{subject index for observation}\;i \end{array}$$

The confidence regression estimates confidence as a function of the two instruments

$$\begin{array}{c} \mu_{i}^{\left(c\right)}=\alpha_{j\left[i\right]}^{\left(c\right)}+\beta_{\text{diff}}\cdot z_{i1}+\beta_{\text{cond}}\cdot z_{i2}\\ c_{i}∼\text{Student}-t\left[\nu_{\text{conf}},\mu_{i}^{\left(c\right)},\sigma_{\text{conf}}\right] \end{array}$$ (13)

The delegate regression estimates delegation as a function of inferred confidence

$$\begin{array}{c} \mu_{i}^{\left(y\right)}=\alpha_{j\left[i\right]}^{\left(y\right)}+\beta_{\text{conf}}\cdot c_{i}\\ y_{i}∼\text{Bernoulli}\left\{{\text{logit}}^{-1}\left[\mu_{i}^{\left(y\right)}\right]\right\} \end{array}$$ (14)

Lastly, we used the following priors for the IV regression model

$$\alpha_{j}^{\left(c\right)}∼t_{3}\left(0,1\right),j=1,\ldots ,J$$

$$\alpha_{j}^{\left(y\right)}∼t_{3}\left(0,1\right),j=1,\ldots ,J$$

$$\beta_{\text{diff}},\beta_{\text{cond}},\beta_{\text{conf}}∼t_{3}\left(0,1\right)$$

$$\sigma_{\text{conf}}∼\text{Exponential}\left(1\right)$$

$$\nu_{\text{conf}}∼\text{Gamma}\left(2,0.1\right)$$

### Fitting DDM

The DDM was fit using the Python package PyDDM (*64*). We fit a collapsing bound according to a step function

$$u\left(t\right)=\text{max}\left(B_{0}-\frac{t}{l}h,0\right)$$ (15)

where u(t) is the bound value at each time point t, $B_{0}$ is the initial value of the bound, l is the time length of the step for collapsing, and h is the height of the step for collapsing.

Drift rate *v* was fit as a linear function of the log-transformed difference in dot numbers between two circles of each trial. We also fit starting-point bias $x_{0}$ and nondecision time *NDT*. PyDDM uses differential evolution to perform the model fitting. All the fitting was performed to each participant’s data for Group/Self condition separately with stimulus coding (Left/Right option). In model fitting, the DDM process is mixed with 5% chance of a uniform distribution that represents responses that came from a non-DDM process.

### Metacognitive process model

There are several existing modeling frameworks on metacognitive modeling. In this study, we adopt the process model in (*28*), which assumes that confidence results from a continuous but noisy and potentially biased transformation of decision values, described by a confidence link function. It can be directly fit to confidence ratings and choices across stimuli of different strengths, both of which are included in our experimental design.

In this model, there are five parameters: (i) The sensory noise parameter represents the noise at the sensory level, which changes the slope of the psychometric function. (ii) The sensory bias parameter captures systematic preferences for one response category, with positive or negative values leading to a propensity to choose one stimulus category or the other. (iii) The metacognitive noise parameter reflects the noise in the transformation from sensory decision values to confidence reports. (iv) The multiplicative evidence bias parameter represents the scaling of absolute sensory decision values. The neutral, unbiased value of the multiplicative evidence bias is 1, and a value less than 1 indicates underconfidence (i.e., the evidence is downscaled), while a value greater than 1 indicates overconfidence (i.e., the evidence is upscaled). (v) The additive evidence bias parameter represents an additive bias such that metacognitive evidence is systematically increased or decreased regardless of the level of sensory evidence. The neutral, unbiased value of the additive evidence bias is 0, and a value less than 0 indicates underconfidence, while a value greater than 0 indicates overconfidence.

We fit the metacognitive model to the participants’ choices and confidence ratings across the stimulus in forced-choice tasks through the model’s python package ReMeta (*28*). We obtained estimated parameters for each condition separately. We assumed that the dominant source of metacognitive noise in our task was in the confidence reporting stage when we configured the model.

### Parameter sensitivity analysis

To test the contribution of each factor (parameter) toward explaining the change in behaviors between Group and Self trials, we used each fitted parameter in Group condition separately combined with the remaining fitted parameters from the Self condition to simulate data. Afterward, we analyzed the simulated data using the same Bayesian hierarchical (generalized) regressions (see Materials and Methods section “Regression analysis”) used for the empirical data to test how well specific parameter changes could account for the observed effects of social responsibility on behavior measures such as RT, accuracy, confidence, and delegation. For factors that consist of two or more interdependent parameters, such as the collapsing bound, we tested the combined effect of all parameters for that factor together.

### Normative model for delegation

#### Framework

We propose that, on every trial, the decision of whether to delegate or make decisions by themselves involves the value comparison between these two actions. To make this decision, the agent computes two action values, one for leading (making decisions by themselves) $Q_{l}$, and one for delegation $Q_{d}$. We fix the reward for the decision as r=1. Thus, $Q_{l}$ is equal to the probability of the individual’s answer being correct, which is approximated by the confidence in their own decisions $c_{\text{Ind}}$. Similarly, for the delegated decision, the reward is influenced by the probability of the delegates’ answer being correct (expert level in our task), which can be approximated by the individuals’ confidence in the others $c_{\text{Others}}$.

Beyond the difference in $Q_{d}$ and $Q_{l}$, the delegation decision may also be determined by a person’s subjective delegation preference, $\phi_{d}$. If $\phi_{d}$ is not equal to zero, the decision to delegate would not be purely based on maximizing the probability of winning the reward. In our model specification, positive values of $\phi_{d}$ lead to a bias for delegation, while negative values represent a bias for leading.

Last, the decision to delegate to others on a given trial is determined by

$$\Delta =Q_{d}-Q_{l}+\phi_{d}$$ (16)

a=f(Δ) (17)

where f() is a step function to convert the difference in *Q* values into the decision of choosing to delegate or lead on each trial.

#### Model fitting

To fit this model, we combine the confidence ratings that were collected in the forced-choice task and the delegate/lead decisions made during the delegation task. Both confidence ratings and delegation decisions may differ for the same decision problem (number and arrangement of dot stimuli) in the same condition due to noise, attention, or other unobserved factors. Therefore, we used the average of the confidence ratings and the proportion of choosing to delegate for each level of stimulus strength within each condition to estimate the model parameters. We then use the logistic function to convert the $\bar{\Delta }$ values into a proportion of choosing to delegate at each stimulus strength. We used a hierarchical Bayesian modeling approach to fit our model using JAGS (*65*), fitting the model to choices from each expert level and condition separately.

#### Mechanisms of delegation preference

There are multiple, nonmutually exclusive, ways in which a delegation preference ($\phi_{d}$) may cause an agent’s behavior to deviate from the normative calculation of $Q_{d}$ minus $Q_{l}$. We tested combinations of the following three possibilities.

1) The delegation preference is a fixed constant regardless of the agent’s confidence levels; for instance, if the agent is predisposed to delegate to others when they are entirely uncertain, they will maintain the same inclination to delegate even when they are entirely certain

$$\phi_{d}=\alpha_{\phi_{d}}$$ (18)

where $\alpha_{\phi_{d}}$ is a constant that does not depend on confidence in self or others.

2) The preference for delegation decreases as the agent’s confidence in themselves increases. In this case, the delegation preference is

$$\phi_{d}=\beta_{\phi_{d}}*c_{\text{Ind}},\beta_{\phi_{d}}<0$$ (19)

where $\beta_{\phi_{d}}$ is the coefficient for the confidence-in-self–dependent effect.

3) The preference for delegation increases when the agent’s confidence in others is higher. In this case, the delegation preference is

$$\phi_{d}=\gamma_{\phi_{d}}*c_{\text{Others}},\gamma_{\phi_{d}}>0$$ (20)

where $\gamma_{\phi_{d}}$ is the coefficient for the confidence-in-others–dependent effect.

We fit three different model specifications to test (i) option 1 alone, (ii) option 2 alone, and (iii) options 1 and 2 combined (i.e., both a constant and confidence-dependent bias). In our task, there are only two levels of confidence in others (accuracy of experts). Therefore, we did not construct models explicitly on the basis of option 3. Instead, we compared the values of $\alpha_{\phi_{d}}$ when fit to trials with different levels of experts to infer whether changing the level of experts altered the delegation preference.

We used the following priors for the model fitting

$$\alpha_{\phi_{d}}∼\text{Uniform}\left(-10,10\right)$$

$$\beta_{\phi_{d}}∼\text{Uniform}\left(0,10\right)$$

#### Model comparison

We used the deviance information criterion (DIC) (*66*) to compare the three different delegation models. The DIC is a hierarchical modeling generalization of the Akaike information criterion. The equation is as follows

$$\text{DIC}=D\left(\bar{\theta }\right)+2p_{D}$$ (21)

where $p_{D}=\frac{1}{2}\bar{\mathrm{Var}\left[D\left(\theta \right)\right]}$. D(θ) is the deviance, for which mean and SD are calculated in JAGS.

### Leave-one-out predictions of the final accuracy in the delegation task

We assessed the prediction performance of linear regression models aimed at predicting final accuracy (the total accuracy of both delegated and led decisions) in the delegation task, using each individual’s estimated absolute delegation preference as the predictor. Specifically, we fit four models, one for each condition (Group, Self) by expert level (70, 90%) combination. These linear regression models were trained to predict final accuracy based on participants’ absolute delegation preferences. For each individual, the model was trained on all other individuals and then used to predict the held-out participant’s final accuracy in that context (e.g., Group trials with 90% experts). Subsequently, we computed the Pearson correlation between the leave-one-out predictions and the true final accuracy values.

## Supplementary Materials

### This PDF file includes:

Supplementary Text Figs. S1 to S7 Tables S1 to S4 References

## References

[1] A. G. Sanfey, Social decision-making: Insights from game theory and neuroscience. Science 318, 598–602 (2007). 17962552 10.1126/science.1142996

[2] B. Bass, R. Bass, *The Bass Handbook of Leadership: Theory, Research, and Managerial Applications* (New York: Free Press, 2009).

[3] Z. Wang, Y. Kuang, H.-Y. Tang, C. Gao, A. Chen, K. Q. Chan, Are decisions made by group representatives more risk averse? The effect of sense of responsibility. J. Behav. Decis. Mak. 31, 311–323 (2018).

[4] D. S. Fareri, J. E. Stasiak, P. Sokol-Hessner, Choosing for others changes dissociable computational mechanisms underpinning risky decision-making. Sci. Rep. 12, 14361 (2022). 35999449 10.1038/s41598-022-18437-9 PMC9399086

[5] G. Charness, M. O. Jackson, The role of responsibility in strategic risk-taking. J. Econ. Behav. Organ. 69, 241–247 (2009).

[6] G. E. Bolton, A. Ockenfels, J. Stauf, Social responsibility promotes conservative risk behavior. Eur. Econ. Rev. 74, 109–127 (2015).

[7] J. Pahlke, S. Strasser, F. M. Vieider, Responsibility effects in decision making under risk. J. Risk Uncertain. 51, 125–146 (2015).

[8] F. M. Vieider, C. Villegas-Palacio, P. Martinsson, M. Mejía, Risk taking for oneself and others: A structural model approach. Econ. Inq. 54, 879–894 (2016).

[9] M. G. Edelson, R. Polania, C. C. Ruff, E. Fehr, T. A. Hare, Computational and neurobiological foundations of leadership decisions. Science 361, eaat0036 (2018). 30072510 10.1126/science.aat0036

[10] S. M. Fleming, R. S. Weil, Z. Nagy, R. J. Dolan, G. Rees, Relating introspective accuracy to individual differences in brain structure. Science 329, 1541–1543 (2010). 20847276 10.1126/science.1191883 PMC3173849

[11] T. O. Nelson, Metamemory: A theoretical framework and new findings. Psychol. Learn. Motiv. 26, 125–173 (1990).

[12] B. De Martino, S. M. Fleming, N. Garrett, R. J. Dolan, Confidence in value-based choice. Nat. Neurosci. 16, 105–110 (2013). 23222911 10.1038/nn.3279 PMC3786394

[13] K. da Silva Castanheira, S. M. Fleming, A. R. Otto, Confidence in risky value-based choice. Psychon. Bull. Rev. 28, 1021–1028 (2021). 33403535 10.3758/s13423-020-01848-y

[14] K. Desender, A. Boldt, N. Yeung, Subjective confidence predicts information seeking in decision making. Psychol. Sci. 29, 761–778 (2018). 29608411 10.1177/0956797617744771

[15] A. Boldt, C. Blundell, B. De Martino, Confidence modulates exploration and exploitation in value-based learning. Neurosci. Conscious. 2019, niz004 (2019). 31086679 10.1093/nc/niz004 PMC6505439

[16] L. Schulz, M. Rollwage, R. J. Dolan, S. M. Fleming, Dogmatism manifests in lowered information search under uncertainty. Proc. Natl. Acad. Sci. U.S.A. 117, 31527–31534 (2020). 33214149 10.1073/pnas.2009641117 PMC7733856

[17] N. Pescetelli, N. Yeung, The role of decision confidence in advice-taking and trust formation. J. Exp. Psychol. Gen. 150, 507–526 (2021). 33001684 10.1037/xge0000960

[18] C. Heyes, D. Bang, N. Shea, C. D. Frith, S. M. Fleming, Knowing ourselves together: The cultural origins of metacognition. Trends Cogn. Sci. 24, 349–362 (2020). 32298621 10.1016/j.tics.2020.02.007 PMC7903141

[19] D. Bang, L. Aitchison, R. Moran, S. Herce Castanon, B. Rafiee, A. Mahmoodi, J. Y. F. Lau, P. E. Latham, B. Bahrami, C. Summerfield, Confidence matching in group decision-making. Nat. Hum. Behav. 1, 0117 (2017).

[20] B. Bahrami, K. Olsen, D. Bang, A. Roepstorff, G. Rees, C. Frith, What failure in collective decision-making tells us about metacognition. Philos. Trans. R. Soc. B Biol. Sci. 367, 1350–1365 (2012). 10.1098/rstb.2011.0420 PMC3318766 22492752

[21] S. Massoni, T. Gajdos, J.-C. Vergnaud, Confidence measurement in the light of signal detection theory. Front. Psychol. 5, 1455 (2014). 25566135 10.3389/fpsyg.2014.01455 PMC4263084

[22] Z. Dienes, A. Seth, Gambling on the unconscious: A comparison of wagering and confidence ratings as measures of awareness in an artificial grammar task. Conscious. Cogn. 19, 674–681 (2010). 19828331 10.1016/j.concog.2009.09.009

[23] G. Hollard, S. Massoni, J.-C. Vergnaud, In search of good probability assessors: An experimental comparison of elicitation rules for confidence judgments. Theory Dec. 80, 363–387 (2016).

[24] E. Karni, A mechanism for eliciting probabilities. Econometrica 77, 603–606 (2009).

[25] G. Malhotra, D. S. Leslie, C. J. H. Ludwig, R. Bogacz, Time-varying decision boundaries: Insights from optimality analysis. Psychon. Bull. Rev. 25, 971–996 (2018). 28730465 10.3758/s13423-017-1340-6 PMC5990589

[26] R. J. Boag, R. J. Innes, N. Stevenson, G. Bahg, J. R. Busemeyer, G. E. Cox, C. Donkin, M. J. Frank, G. E. Hawkins, A. Heathcote, C. Hedge, V. Lerche, S. D. Lilburn, G. D. Logan, D. Matzke, S. Miletić, A. F. Osth, T. J. Palmeri, P. B. Sederberg, H. Singmann, P. L. Smith, T. Stafford, M. Steyvers, L. Strickland, J. S. Trueblood, K. Tsetsos, B. M. Turner, M. Usher, L. van Maanen, D. van Ravenzwaaij, J. Vandekerckhove, A. Voss, E. R. Weichart, G. Weindel, C. N. White, N. J. Evans, S. D. Brown, B. U. Forstmann, An expert guide to planning experimental tasks for evidence-accumulation modeling. Adv. Methods Pract. Psychol. Sci. 8, 25152459251336127 (2025).

[27] R. Ratcliff, A. Thapar, P. Gomez, G. McKoon, A diffusion model analysis of the effects of aging in the lexical-decision task. Psychol. Aging 19, 278–289 (2004). 15222821 10.1037/0882-7974.19.2.278 PMC1360155

[28] M. Guggenmos, Reverse engineering of metacognition. eLife 11, e75420 (2022). 36107147 10.7554/eLife.75420 PMC9477496

[29] N. Haddara, D. Rahnev, The impact of feedback on perceptual decision-making and metacognition: Reduction in bias but no change in sensitivity. Psychol. Sci. 33, 259–275 (2022). 35100069 10.1177/09567976211032887 PMC9096460

[30] M. Rouault, P. Dayan, S. M. Fleming, Forming global estimates of self-performance from local confidence. Nat. Commun. 10, 1141 (2019). 30850612 10.1038/s41467-019-09075-3 PMC6408496

[31] R. Frey, A. Pedroni, R. Mata, J. Rieskamp, R. Hertwig, Risk preference shares the psychometric structure of major psychological traits. Sci. Adv. 3, e1701381 (2017). 28983511 10.1126/sciadv.1701381 PMC5627985

[32] G. L. Mazza, H. L. Smyth, P. G. Bissett, J. R. Canning, I. W. Eisenberg, A. Z. Enkavi, O. Gonzalez, S. J. Kim, S. A. Metcalf, F. Muniz, W. E. Pelham III, E. A. Scherer, M. J. Valente, H. Xie, R. A. Poldrack, L. A. Marsch, D. MacKinnon, Correlation database of 60 cross-disciplinary surveys and cognitive tasks assessing self-regulation. J. Pers. Assess. 103, 238–245 (2021). 32148088 10.1080/00223891.2020.1732994 PMC7483539

[33] P. Atanasov, *Risk preferences in choices for self and others: Meta analysis and research directions* (SSRN, 2015); 10.2139/ssrn.1682569.

[34] A. Voodla, A. Uusberg, K. Desender, Metacognitive confidence and affect - two sides of the same coin? Cogn. Emot. 39, 1857–1874 (2025). 39831796 10.1080/02699931.2025.2451795

[35] T. Sharot, M. Rollwage, C. R. Sunstein, S. M. Fleming, Why and when beliefs change. Perspect. Psychol. Sci. 18, 142–151 (2023). 35939828 10.1177/17456916221082967

[36] M. Rouault, T. Seow, C. M. Gillan, S. M. Fleming, Psychiatric symptom dimensions are associated with dissociable shifts in metacognition but not task performance. Biol. Psych. 84, 443–451 (2018). 10.1016/j.biopsych.2017.12.017 PMC6117452 29458997

[37] M. Lebreton, S. Langdon, M. J. Slieker, J. S. Nooitgedacht, A. E. Goudriaan, D. Denys, R. J. van Holst, J. Luigjes, Two sides of the same coin: Monetary incentives concurrently improve and bias confidence judgments. Sci. Adv. 4, eaaq0668 (2018). 29854944 10.1126/sciadv.aaq0668 PMC5976269

[38] M. Lebreton, K. Bacily, S. Palminteri, J. B. Engelmann, Contextual influence on confidence judgments in human reinforcement learning. PLOS Comput. Biol. 15, e1006973 (2019). 30958826 10.1371/journal.pcbi.1006973 PMC6472836

[39] C.-C. Ting, N. Salem-Garcia, S. Palminteri, J. B. Engelmann, M. Lebreton, Neural and computational underpinnings of biased confidence in human reinforcement learning. Nat. Commun. 14, 6896 (2023). 37898640 10.1038/s41467-023-42589-5 PMC10613217

[40] M. Sherif, *A Study of Some Social Factors in Perception* (Archives of Psychology, Columbia University, 1935).

[41] S. E. Ash, “Effects of group pressure upon the modification and distortion of judgements,” in *Groups, Leadership and Men; Research in Human Relations*, H. Guetzkow Ed. (Carnegie Press, 1951), pp. 177–190. https://psycnet.apa.org/record/1952-00803-001.

[42] R. Bond, P. B. Smith, Culture and conformity: A meta-analysis of studies using Asch’s (1952b, 1956) line judgment task. Psychol. Bull. 119, 111–137 (1996).

[43] R. Bond, Group size and conformity. GPIR 8, 331–354 (2005).

[44] U. Toelch, F. Panizza, H. R. Heekeren, Norm compliance affects perceptual decisions through modulation of a starting point bias. R. Soc. Open Sci. 5, 171268 (2018). 29657747 10.1098/rsos.171268 PMC5882671

[45] M. Germar, V. H. Duderstadt, A. Mojzisch, Social norms shape visual appearance: Taking a closer look at the link between social norm learning and perceptual decision-making. Cognition 241, 105611 (2023). 37678084 10.1016/j.cognition.2023.105611

[46] A. Mahmoodi, K. Ringwald, M. K. Wittmann, C. Mehring, Social context alters metacognition. PsyArXiv [Preprint] (2019). 10.31234/osf.io/ez8qw.

[47] N. Trudel, P. L. Lockwood, M. F. Rushworth, M. K. Wittmann, Neural activity tracking identity and confidence in social information. eLife 12, e71315 (2023). 36763582 10.7554/eLife.71315 PMC9917428

[48] L. Schooler, M. Okhan, S. Hollander, M. Gill, Y. Zoh, M. J. Crockett, H. Yu, Confidence in moral decision-making. Collabra Psychol. 10, 121387 (2024).

[49] M. Rouault, S. M. Fleming, Formation of global self-beliefs in the human brain. Proc. Natl. Acad. Sci. U.S.A. 117, 27268–27276 (2020). 33060292 10.1073/pnas.2003094117 PMC7959580

[50] A. McWilliams, H. Bibby, N. Steinbeis, A. S. David, S. M. Fleming, Age-related decreases in global metacognition are independent of local metacognition and task performance. Cognition 235, 105389 (2023). 36764048 10.1016/j.cognition.2023.105389 PMC10632679

[51] A. Bandura, Self-efficacy: Toward a unifying theory of behavioral change. Adv. Behav. Res. Ther. 1, 139–161 (1978). 10.1037//0033-295x.84.2.191 847061

[52] R. Elliott, B. J. Sahakian, A. McKay, J. J. Herrod, T. W. Robbins, E. S. Paykel, Neuropsychological impairments in unipolar depression: The influence of perceived failure on subsequent performance. Psychol. Med. 26, 975–989 (1996). 8878330 10.1017/s0033291700035303

[53] G. Zacharopoulos, N. Binetti, V. Walsh, R. Kanai, The effect of self-efficacy on visual discrimination sensitivity. PLOS ONE 9, e109392 (2014). 25295529 10.1371/journal.pone.0109392 PMC4190082

[54] H. Van Marcke, P. L. Denmat, T. Verguts, K. Desender, Manipulating prior beliefs causally induces under- and overconfidence. Psychol. Sci. 35, 358–375 (2024). 38427319 10.1177/09567976241231572

[55] R. van den Berg, A. Zylberberg, R. Kiani, M. N. Shadlen, D. M. Wolpert, Confidence is the bridge between multi-stage decisions. Curr. Biol. 26, 3157–3168 (2016). 27866891 10.1016/j.cub.2016.10.021 PMC5154755

[56] E. Schulz, S. J. Gershman, The algorithmic architecture of exploration in the human brain. Curr. Opin. Neurobiol. 55, 7–14 (2019). 30529148 10.1016/j.conb.2018.11.003

[57] J. Gottlieb, P.-Y. Oudeyer, Towards a neuroscience of active sampling and curiosity. Nat. Rev. Neurosci. 19, 758–770 (2018). 30397322 10.1038/s41583-018-0078-0

[58] L. Schulz, S. M. Fleming, P. Dayan, Metacognitive computations for information search: Confidence in control. Psychol. Rev. 130, 604–639 (2023). 36757948 10.1037/rev0000401

[59] P. Gerrans, *The Measure of Madness: Philosophy of Mind, Cognitive Neuroscience, and Delusional Thought* (MIT Press, 2014); 10.7551/mitpress/9780262027557.001.0001.

[60] T. L. Griffiths, F. Lieder, N. D. Goodman, Rational use of cognitive resources: Levels of analysis between the computational and the algorithmic. Top. Cogn. Sci. 7, 217–229 (2015). 25898807 10.1111/tops.12142

[61] C. C. Eckel, P. J. Grossman, Managing diversity by creating team identity. J. Econ. Behav. Org. 58, 371–392 (2005).

[62] P.-C. Bürkner, brms: An R package for bayesian multilevel models using stan. J. Stat. Softw. 80, 1–28 (2017).

[63] R Core Team, *R: A Language and Environment for Statistical Computing* (R Foundation for Statistical Computing, Vienna, Austria, 2021).

[64] M. Shinn, N. H. Lam, J. D. Murray, A flexible framework for simulating and fitting generalized drift-diffusion models. eLife 9, e56938 (2020). 32749218 10.7554/eLife.56938 PMC7462609

[65] M. Plummer, JAGS: A program for analysis of Bayesian graphical models using Gibbs sampling, in *Proceedings of the 3rd International Workshop on Distributed Statistical Computing* (Vienna, Austria, 2003), vol. 124, pp. 1–10.

[66] A. Gelman, J. B. Carlin, H. S. Stern, D. B. Rubin, Eds., *Bayesian Data Analysis*, *Texts in Statistical Science* (Chapman & Hall/CRC, 2003); 10.1201/9780429258480.

[67] E. Holmes, P. T. Kitterick, A. Q. Summerfield, Cueing listeners to attend to a target talker progressively improves word report as the duration of the cue-target interval lengthens to 2,000 ms. Atten. Percept. Psychophys. 80, 1520–1538 (2018). 29696570 10.3758/s13414-018-1531-x

[68] S. D. Brown, A. Heathcote, The simplest complete model of choice response time: Linear ballistic accumulation. Cogn. Psychol. 57, 153–178 (2008). 18243170 10.1016/j.cogpsych.2007.12.002

[69] L. Fontanesi, laurafontanesi/rlssm: First dev release (2021); 10.5281/zenodo.4562217.

[70] A. Vehtari, A. Gelman, J. Gabry, Practical Bayesian model evaluation using leave-one-out cross-validation and WAIC. Stat. Comput. 27, 1413–1432 (2017).

[71] T. Sivula, M. Magnusson, A. A. Matamoros, A. Vehtari, Uncertainty in Bayesian leave-one-out cross-validation based model comparison. 10.48550/arXiv.2008.10296 (2025).

