# Supplementary material for: Deciding for others alters metacognition leading to responsibility aversion
Source: Sci Adv. 2026 Feb 25;12(9):eady0441. doi: 10.1126/sciadv.ady0441 (PMC12935019; doi:10.1126/sciadv.ady0441)
Supplement: Supplementary file 1 — Supplementary Text Figs. S1 to S7 Tables S1 to S4 References [file sciadv.ady0441_sm.pdf]

Science Advances
AAAS

Supplementary Materials for

Deciding for others alters metacognition leading to responsibility aversion

Sherry Dongqi Bao *et al.*

Corresponding author: Sherry Dongqi Bao, dongqi.bao@econ.uzh.ch; Todd A. Hare, todd.hare@econ.uzh.ch

Sci. Adv. **12**, eady0441 (2026)
DOI: 10.1126/sciadv.ady0441

This PDF file includes:

Supplementary Text
Figs. S1 to S7
Tables S1 to S4
References

Supplementary Text

Correlations between confidence, accuracy, and RT measures

Changes in confidence significantly correlate with changes in RT, but not accuracy. Lower confidence under social responsibility was not consistently correlated with lower accuracy at the individual level across tasks (Spearman's rank correlation in the limited display task: $\rho = 0.2$, $P = 0.02$, Figure S1A; in the unlimited display task: $\rho = 0.12$, $P = 0.42$, Figure S1B). However, the decrease in confidence under social responsibility was strongly correlated with longer RTs for both the task with limited display time (Figure S1C; Spearman's rank correlation $\rho = -0.47$, $P < 0.001$) and the task with unlimited display time (Figure S1D; Spearman's rank correlation $\rho = -0.457$, $P < 0.001$). These results suggest that lower confidence under social responsibility cannot be explained by worse performance, but may be related to processes that lead to longer RTs. However, the association between confidence and RT effects was not found in the version of the experiment that provided feedback on performance, indicating that the two effects are at least partially independent.

Effects of social responsibility on confidence rating times and stimulus initiation times

Participants took longer to rate their confidence in the Group versus Self condition (Figure S5A-B). This difference was large in the unlimited-display task (Equation S1; main effect of condition $\beta_{S1.1}$=0.03, 95% CI = [0.011, 0.048], $P_{MCMC} < 0.001$). Although the confidence-rating-time difference in the limited display time task went in the same direction, it was less robust (main effect of condition $\beta_{S1.1}$=0.01, 95% CI = [-0.0029, 0.0190], $P_{MCMC} = 0.08$).

Stimulus-initiation times were slower under responsibility for others too. Participants had to press a button to initiate the dots display at the beginning of each trial. Inspired by previous reports that task performance improves as the duration of the cue-target interval increases (*67*), we computed the stimulus-initiation time as the difference between the display of the trial onset (empty circles) and the button press to trigger the dot displays inside the circles, which is similar to the cue-target interval. We found that people took longer to initiate the stimulus when responsible for others both in the limited (Equation S2; main effect of condition $\beta_{S2.1}$=0.06, 95% CI = [0.045, 0.083], $P_{MCMC} < 0.001$) and unlimited (main effect of condition $\beta_{S2.1}$=0.04, 95% CI = [0.012, 0.076], P_{MCMC} <

0.001) display time experiments, as shown in Figure S5C-D. Participants who spent a longer time to initiate stimulus also tended to take a longer time to respond in Group compared to Self trials (Figure S6), which suggests the effects of social responsibility may be related at the two phases. In summary, participants were slower on all response-time measures (trial initiation, decisions, and confidence ratings) when they were responsible for determining others' payoffs.

The regression testing how confidence rating and Group/Self condition were related to confidence rating time is listed below:

$$\text{confidenceLogRT} = \alpha_{S1} + \beta_{S1.1}\text{Condition} + \beta_{S1.2}\text{Confidence} + \beta_{S1.3}\text{ConfidenceSquared} + e \quad \text{(S1)}$$

Here, confidenceLogRT is the log-transformed confidence rating time in each trial, ConfidenceSquared is the square of the standardized confidence rating.

The regression testing how Group/Self condition was related to stimulus initiation time is as follows:

$$\text{initiationRT} = \alpha_{S2} + \beta_{S2.1}\text{Condition} + e \quad \text{(S2)}$$

Here, initiationRT is the log-transformed stimulus initiation time in each trial.

Testing decision mechanisms underlying behavior changes with social responsibility using LBA

Following an anonymous reviewer's suggestion to test if our results are robust across different modeling approaches, we fit the data using an alternative sequential sampling model—the Linear Ballistic Accumulator (LBA) (*68*). The LBA framework differs from the DDM in that it uses linear, deterministic, and independent accumulators, as well as a constant boundary. Similar to the DDM, LBA models have been shown to effectively capture behavior in both binary and multi-option choice tasks (*68*).

In our implementation, the LBA model included four free parameters: the starting point interval (*SPI*, reflecting variability in initial evidence), distance to bound (*DTB*, reflecting decision threshold), non-decision time (*NDT*), and drift scaling (*Drift*). The drift rate for each accumulator was proportional to the product of the drift scaling parameter and the log-transformed number of dots in each circle. The LBA model was fit using modified code based on the Python package *rlssm* (*69*).

After fitting, we conducted parameter sensitivity analyses to test whether changes in specific parameters were sufficient to reproduce the effects observed in the empirical data (Figure S7). We found that simulations using the full set of fitted parameters for each condition successfully reproduced the effects of social responsibility on both decision RTs and accuracy. Again, no single parameter was sufficient to account for the observed differences in RT and accuracy between conditions, which is consistent with the conclusions we reached using the DDM with collapsing bounds.

Metacognitive biases relate to individual confidence changes

Although there is a significant decrease in confidence between the Self and Group trials, on average, there is also substantial individual variability in the size of the confidence change. We tested whether this variability is linked to individual (meta)cognitive parameters as derived from the metacognitive model. We built a linear regression model (Equation S3) to predict changes in confidence based on the average values of fitted (meta)cognitive parameters (sensory noise, sensory bias, metacognitive noise, metacognitive multiplicative bias, and metacognitive additive bias) in both the Group and Self conditions. We also included the absolute values of the metacognitive multiplicative and additive biases to represent the extent of bias. Our findings indicate that higher levels of metacognitive multiplicative bias (further away from being 1, ($\beta_{S3.5}$=0.13, 95% CI = [0.02, 0.25], P_{MCMC} = 0.01)) and greater metacognitive additive bias (further away from 0, $\beta_{S3.7}$=0.15, 95% CI = [0.03, 0.28], P_{MCMC} = 0.01) are associated with a smaller reduction in confidence when moving from the Self to the Group condition. In other words, social responsibility does not exaggerate metacognitive biases as much in people who are poorly calibrated to begin with. Note that this result may be driven by a floor effect in terms of how poorly calibrated a healthy adult can be in our magnitude judgment task. Thus, we should interpret this result with caution.

The regression testing how the fitted (meta)cognitive parameters from the metacognitive model

were related to confidence change is listed below:

$$\begin{aligned}\text{ConfidenceChange} = \alpha_{S3} &+ \beta_{S3.1}\text{SensoryNoise} + \beta_{S3.2}\text{SensoryBias} + \beta_{S3.3}\text{MetacognitiveNoise} \\ &+ \beta_{S3.4}\text{MetacognitiveMultiplicativeBias} + \beta_{S3.5}\left|\text{MetacognitiveMultiplicativeBias} - 1\right| \\ &+ \beta_{S3.6}\text{MetacognitiveAdditiveBias} + \beta_{S3.7}\left|\text{MetacognitiveAdditiveBias}\right|\end{aligned} \tag{S3}$$

In this equation, all the regressors and ConfidenceChange are rank transformed.

Responsibility aversion and final accuracy

Responsibility aversion does not lead to better final accuracy. As evident from Figure S4B, most participants' ϕ_d is smaller than 0 in both Group and Self conditions and with both levels of experts. In other words, participants exhibit a bias towards leading too often in our task. Given that people exhibit responsibility aversion in the Group condition, and are thus less biased toward leading, one could predict that the increase in delegation proportion will increase the final accuracy in that condition. However, the correlations between individual changes in delegation proportion and final accuracy between the Group and Self conditions in the Experts 70% condition (Spearman's rank correlation $\rho = 0.097$, $P = 0.15$) and the Experts 90% condition (Spearman's rank correlation $\rho = -0.034$, $P = 0.64$) were not significant. This is because the range of stimulus strength where responsibility aversion occurs plays an important role as well. Figure 8A-B displays the increase in delegation proportion between the Group and Self conditions across differences in the number of dots between two circles and experts' accuracy levels. At both expert levels, participants sometimes increased their delegation proportions even in trials where their accuracy exceeded that of the experts, while at other times they did not increase delegation despite performing below the experts' level. This makes the effect of responsibility aversion on final accuracy mixed, yielding no net benefit or cost in this specific context.

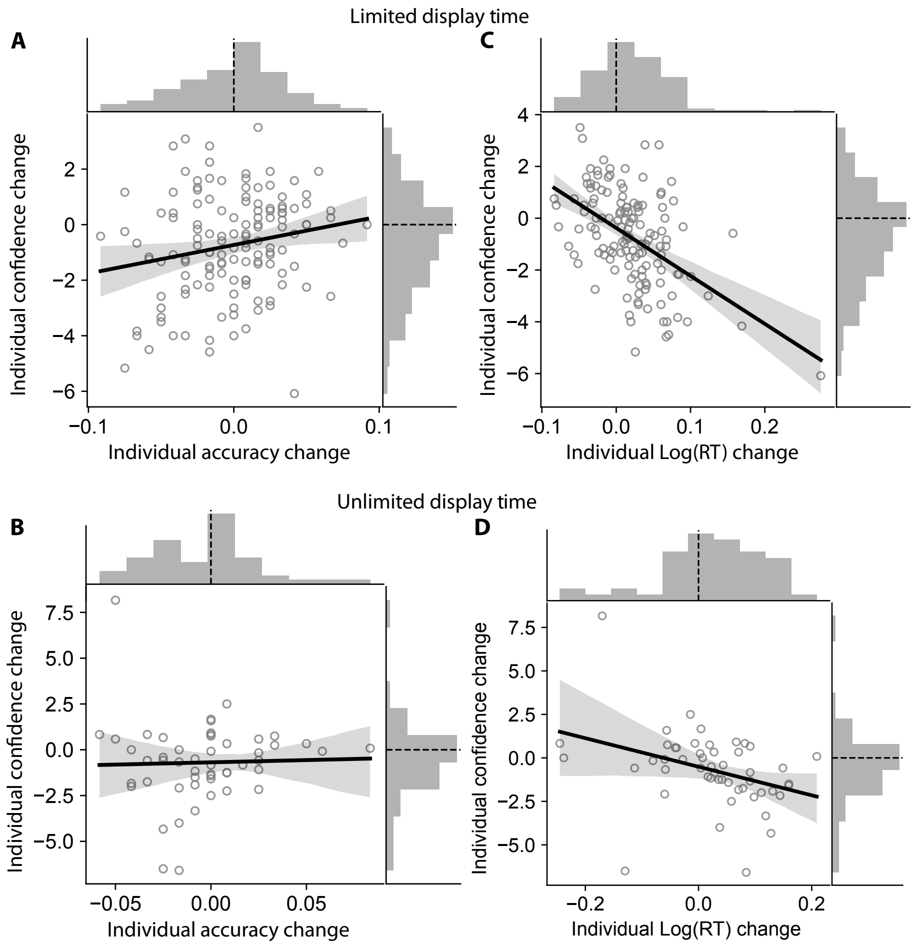

Figure S1: **Correlations between individual changes in confidence, accuracy, and RT across Group and Self conditions in limited and unlimited display time experiments.** Each column represents a measurement type, while each row denotes an experiment version. Grey shading represents 95% confidence interval for the regression estimate. (**A-B**) The correlation of individual average change in confidence and change in accuracy between two conditions (Group - Self). In limited display task: Spearman's rank correlation $\rho = 0.2$, $P = 0.02$; in unlimited display task: $\rho = 0.12, P = 0.42$. (**C-D**) The correlation of individual average change in confidence and change in log-transformed RT between the two conditions (Group - Self). In limited display task: $\rho = -0.47$, $P < 0.001$; in unlimited display task: $\rho = -0.457$, $P < 0.001$.

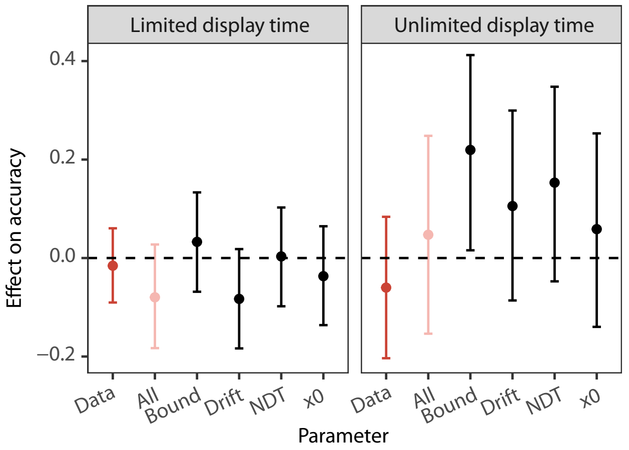

Figure S2: **The error bar plots show the result of a parameter sensitivity analysis that tested whether responsibility induced changes to specific subsets of the parameters in the DDM can reproduce the effects observed in the behavioral data.** Here, the effect of interest on the y-axis is the influence of the Group condition on accuracy, estimated from the Bayesian hierarchical generalized regression in Equation 1. The layout is the same as Figure 3, but for effect of accuracy.

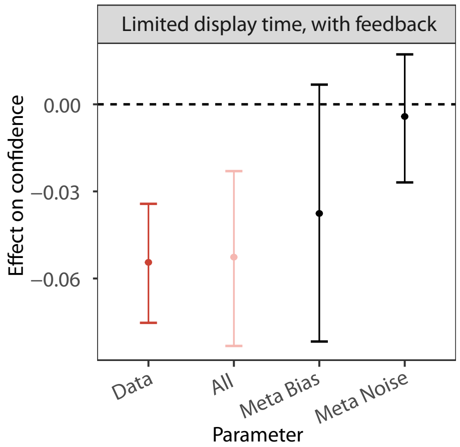

Figure S3: **The error bar plots show the result of a parameter sensitivity analysis that tested whether responsibility induced changes to specific subsets of the parameters in the metacognitive model can reproduce the effects observed in the behavioral data.** The layout is the same as Figure 4, but with the data from the experiment with performance feedback.

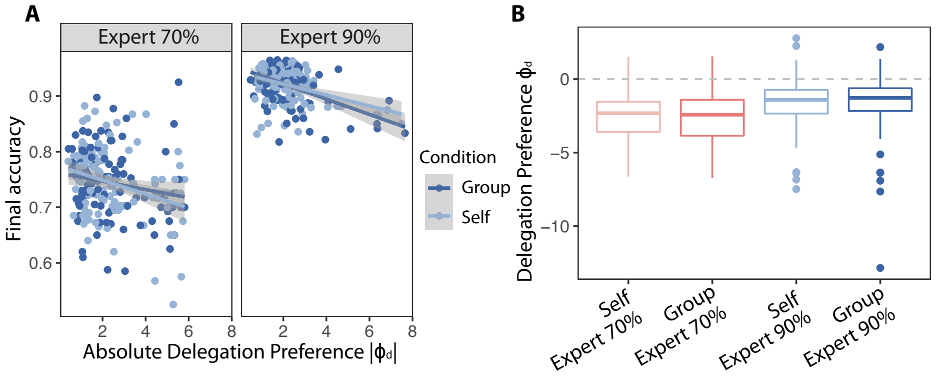

Figure S4: **Delegation preferences and their relationship to final accuracy across conditions and expertise levels.** (**A**) Negative correlations between individual absolute delegation preference and final accuracy in both Group and Self conditions across expert levels. (**B**) Box plot illustrating estimated delegation preference from model fitting across Group and Self conditions and expert levels.

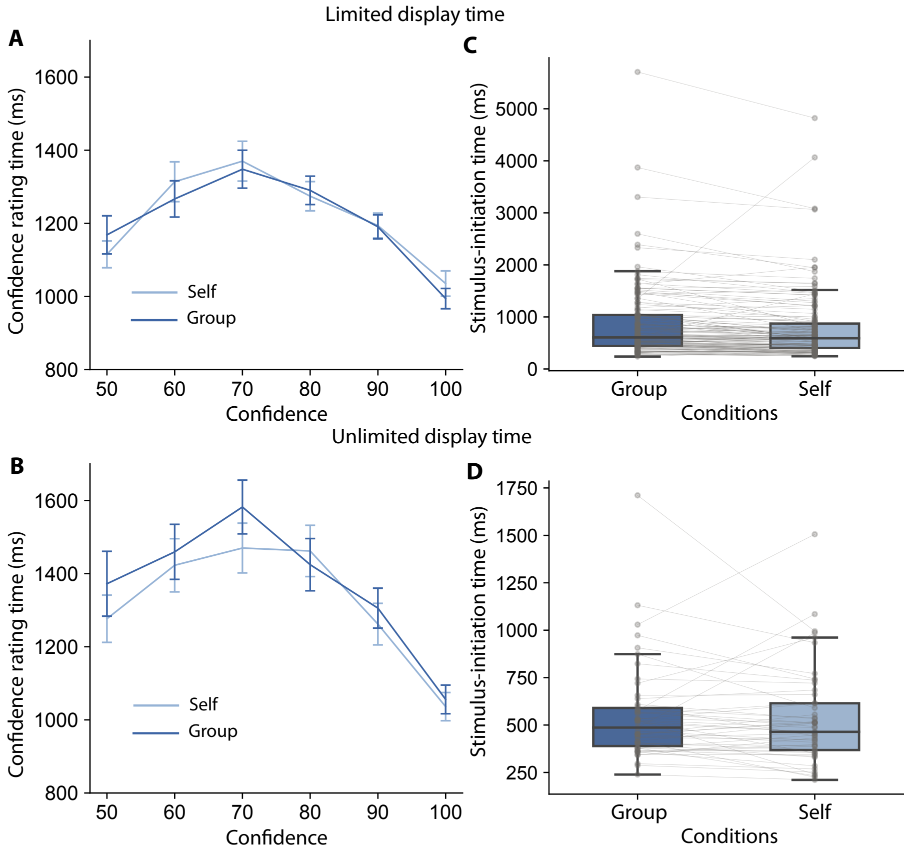

Figure S5: **Confidence rating time and stimulus-initiation time in Group and Self conditions in limited and unlimited display time experiments.** Each column represents a measurement type, while each row denotes an experiment version. (**A-B**) Participants spent a longer time rating confidence when they were responsible for others compared to deciding only for themselves in unlimited-display task. The error bars represent standard error. (**C-D**) Participants spent a longer time before initiating the stimulus when they were responsible for others compared to deciding only for themselves, both in limited-display and unlimited-display tasks. The box extends from the first quartile to the third quartile of the data, with a line at the median. The whiskers extend from the box to the farthest data point lying within 1.5 × the inter-quartile range (IQR) from the box. Each grey dot represents the average stimulus-initiation time for each participant, with the grey lines connecting the same participant across the two conditions.

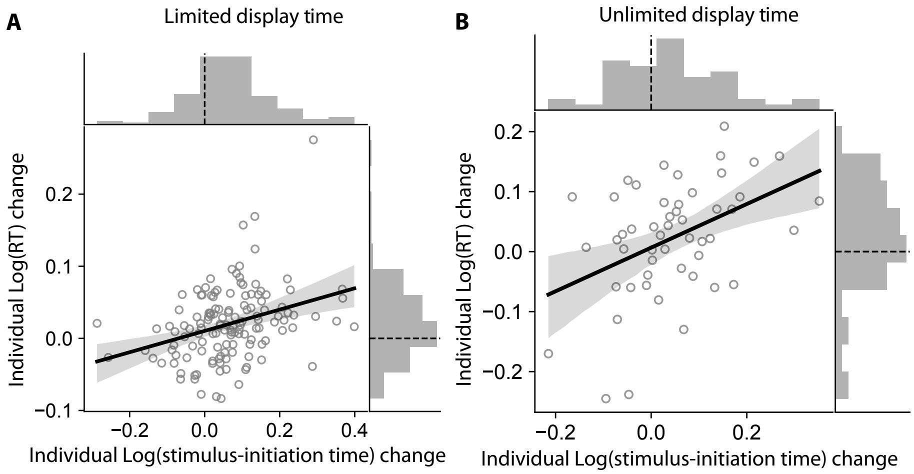

Figure S6: **Participants who spent a longer time to initiate stimulus also tended to take a longer time to respond in Group compared to Self trials.** Grey shading represents 95% confidence interval for the regression estimate. (**A**) Limited display time: Spearman's rank correlation ρ = 0.334, P < 0.001. (**B**) Unlimited display time: ρ = 0.395, P = 0.004.

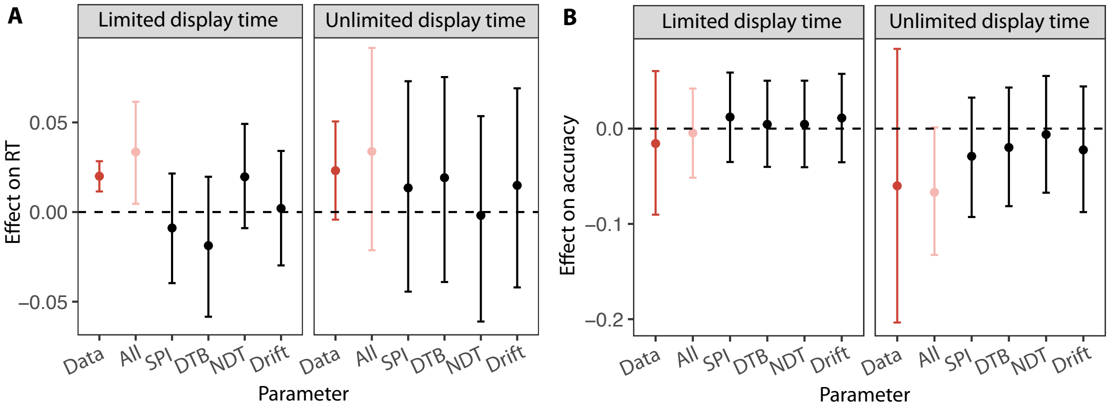

Figure S7: **The error bar plots show the result of a parameter sensitivity analysis that tested whether responsibility induced changes to specific subsets of the parameters in the LBA can reproduce the effects observed in the behavioral data.** (**A**) The effect of interest on the y-axis is the influence of the Group condition on RT, estimated from the Bayesian hierarchical generalized regression in Equation 3. (**B**) The effect of interest on the y-axis is the influence of the Group condition on accuracy, estimated from the Bayesian hierarchical generalized regression in Equation 1.

Table S1: **Confidence regression model comparison based on ELPD (Expected Log Pointwise Predictive Density).** The model comparison is implemented using the “loo” package in R (*70*), which calculates the Bayesian leave-one-out estimate of the expected log pointwise predictive density (“elpd_loo”). The model comparison computes values of “elpd_diff” (the difference in “elpd_loo” between two models) and ”se_diff” (the standard error of the component-wise differences in “elpd_loo” between two models) by performing pairwise comparisons between each model and the model with the largest ELPD (the model in the first row of the model comparison table, also the winning model). Here, “Model 0” is built based on Equation 2, and “Model 1” additionally includes the interaction between confidence and difficulty level in addition to the main effects included in “Model 0”. There is no significant difference between the two models, indicating that the interaction term does not provide a better fit to the data (*71*).

(A) Limited display time task

Models	elpd_diff	se_diff
Model 1	0.0	0.0
Model 0	-1.2	1.6

(B) Unlimited display time task

Models	elpd_diff	se_diff
Model 0	0.0	0.0
Model 1	-1.0	0.3

(C) Task with feedback

Models	elpd_diff	se_diff
Model 0	0.0	0.0
Model 1	-0.8	0.6

Table S2: **Tables of fitted DDM parameters for limited and unlimited display time tasks.** The drift-diffusion model (DDM) with collapsing bound includes six fitted parameters: v (drift rate); for collapsing bounds: B_0 (initial bound height), l (step duration), and h (step height); x_0 (starting-point bias); and NDT (non-decision time). For each parameter, we report the median, mean, and standard deviation (std) across participants.

(A) Limited display time task

	Self Condition			Group Condition		
	Median	Mean	std	Median	Mean	std
v	0.704	0.728	0.211	0.691	0.704	0.191
B_0	1.180	1.641	1.123	1.216	1.644	1.079
l	0.436	0.578	0.482	0.425	0.594	0.534
h	0.209	0.219	0.121	0.187	0.223	0.136
x_0	-0.019	-0.028	0.127	-0.024	-0.011	0.137
NDT	0.581	0.569	0.111	0.616	0.576	0.109

(B) Unlimited display time task

	Self Condition			Group Condition		
	Median	Mean	std	Median	Mean	std
v	0.391	0.423	0.158	0.392	0.419	0.139
B_0	1.978	2.144	0.832	1.967	2.160	0.799
l	1.245	1.261	0.431	1.393	1.336	0.405
h	0.132	0.138	0.103	0.128	0.132	0.100
x_0	0.017	0.006	0.134	0.028	0.020	0.145
NDT	0.461	0.502	0.222	0.468	0.541	0.232

Table S3: **Tables of fitted metacognitive model parameters for limited display time task, unlimited display time task and task with feedback.** Detailed parameter definitions are provided in the Methods section. For each parameter, we report the median, mean, and standard deviation (std) across participants.

(A) Limited display time task

	Self Condition			Group Condition		
	Median	Mean	std	Median	Mean	std
Sensory noise	0.312	0.334	0.090	0.326	0.337	0.087
Sensory bias	0.057	0.045	0.145	0.039	0.055	0.140
Metacognitive noise	0.079	0.106	0.089	0.075	0.100	0.085
Metacognitive multiplicative bias	0.754	0.896	0.631	0.682	0.763	0.507
Metacognitive additive bias	-0.005	-0.043	0.182	-0.009	-0.015	0.135

(B) Unlimited display time task

	Self Condition			Group Condition		
	Median	Mean	std	Median	Mean	std
Sensory noise	0.308	0.342	0.172	0.295	0.337	0.156
Sensory bias	0.062	0.070	0.145	0.096	0.090	0.133
Metacognitive noise	0.130	0.136	0.098	0.122	0.136	0.100
Metacognitive multiplicative bias	0.460	0.868	1.385	0.438	1.278	5.298
Metacognitive additive bias	0.037	0.007	0.326	0.035	0.053	0.284

(C) Task with feedback

	Self Condition			Group Condition		
	Median	Mean	std	Median	Mean	std
Sensory noise	0.304	0.315	0.080	0.321	0.328	0.088
Sensory bias	0.065	0.046	0.144	0.078	0.062	0.146
Metacognitive noise	0.050	0.067	0.069	0.044	0.066	0.074
Metacognitive multiplicative bias	0.957	1.906	4.999	0.981	1.602	2.219
Metacognitive additive bias	0.028	-0.014	0.305	0.026	-0.025	0.302

Table S4: **Delegation model comparison based on Deviance Information Criterion (DIC).** Lower DIC values indicate a better model in terms of the trade-off between model fit and complexity. The comparison includes three different versions of the delegation model fit to either the expert 90% or expert 70% trials: "Constant term" model, which includes one parameter α_{ϕ_d}; "Confidence-dependent term" model, which includes one confidence-dependent coefficient β_{ϕ_d}; "Both terms" model, which includes both parameters α_{ϕ_d} and β_{ϕ_d}.

Expert condition	Both terms	Confidence-dependent term	Constant term
Expert 90%	10287.00	29608.51	17935.17
Expert 70%	28786.77	74166.94	54728.92

REFERENCES

1. A. G. Sanfey, Social decision-making: Insights from game theory and neuroscience. *Science* **318**, 598–602 (2007).

2. B. Bass, R. Bass, *The Bass Handbook of Leadership: Theory, Research, and Managerial Applications* (New York: Free Press, 2009).

3. Z. Wang, Y. Kuang, H.-Y. Tang, C. Gao, A. Chen, K. Q. Chan, Are decisions made by group representatives more risk averse? The effect of sense of responsibility. *J. Behav. Decis. Mak.* **31**, 311–323 (2018).

4. D. S. Fareri, J. E. Stasiak, P. Sokol-Hessner, Choosing for others changes dissociable computational mechanisms underpinning risky decision-making. *Sci. Rep.* **12**, 14361 (2022).

5. G. Charness, M. O. Jackson, The role of responsibility in strategic risk-taking. *J. Econ. Behav. Organ.* **69**, 241–247 (2009).

6. G. E. Bolton, A. Ockenfels, J. Stauf, Social responsibility promotes conservative risk behavior. *Eur. Econ. Rev.* **74**, 109–127 (2015).

7. J. Pahlke, S. Strasser, F. M. Vieider, Responsibility effects in decision making under risk. *J. Risk Uncertain.* **51**, 125–146 (2015).

8. F. M. Vieider, C. Villegas-Palacio, P. Martinsson, M. Mejía, Risk taking for oneself and others: A structural model approach. *Econ. Inq.* **54**, 879–894 (2016).

9. M. G. Edelson, R. Polania, C. C. Ruff, E. Fehr, T. A. Hare, Computational and neurobiological foundations of leadership decisions. *Science* **361**, eaat0036 (2018).

10. S. M. Fleming, R. S. Weil, Z. Nagy, R. J. Dolan, G. Rees, Relating introspective accuracy to individual differences in brain structure. *Science* **329**, 1541–1543 (2010).

11. T. O. Nelson, Metamemory: A theoretical framework and new findings. *Psychol. Learn. Motiv.* **26**, 125–173 (1990).

12. B. De Martino, S. M. Fleming, N. Garrett, R. J. Dolan, Confidence in value-based choice. *Nat. Neurosci.* **16**, 105–110 (2013).

13. K. da Silva Castanheira, S. M. Fleming, A. R. Otto, Confidence in risky value-based choice. *Psychon. Bull. Rev.* **28**, 1021–1028 (2021).

14. K. Desender, A. Boldt, N. Yeung, Subjective confidence predicts information seeking in decision making. *Psychol. Sci.* **29**, 761–778 (2018).

15. A. Boldt, C. Blundell, B. De Martino, Confidence modulates exploration and exploitation in value-based learning. *Neurosci. Conscious.* **2019**, niz004 (2019).

16. L. Schulz, M. Rollwage, R. J. Dolan, S. M. Fleming, Dogmatism manifests in lowered information search under uncertainty. *Proc. Natl. Acad. Sci. U.S.A.* **117**, 31527–31534 (2020).

17. N. Pescetelli, N. Yeung, The role of decision confidence in advice-taking and trust formation. *J. Exp. Psychol. Gen.* **150**, 507–526 (2021).

18. C. Heyes, D. Bang, N. Shea, C. D. Frith, S. M. Fleming, Knowing ourselves together: The cultural origins of metacognition. *Trends Cogn. Sci.* **24**, 349–362 (2020).

19. D. Bang, L. Aitchison, R. Moran, S. Herce Castanon, B. Rafiee, A. Mahmoodi, J. Y. F. Lau, P. E. Latham, B. Bahrami, C. Summerfield, Confidence matching in group decision-making. *Nat. Hum. Behav.* **1**, 0117 (2017).

20. B. Bahrami, K. Olsen, D. Bang, A. Roepstorff, G. Rees, C. Frith, What failure in collective decision-making tells us about metacognition. *Philos. Trans. R. Soc. B Biol. Sci.* **367**, 1350–1365 (2012).

21. S. Massoni, T. Gajdos, J.-C. Vergnaud, Confidence measurement in the light of signal detection theory. *Front. Psychol.* **5**, 1455 (2014).

22. Z. Dienes, A. Seth, Gambling on the unconscious: A comparison of wagering and confidence ratings as measures of awareness in an artificial grammar task. *Conscious. Cogn.* **19**, 674–681 (2010).

23. G. Hollard, S. Massoni, J.-C. Vergnaud, In search of good probability assessors: An experimental comparison of elicitation rules for confidence judgments. *Theory Dec.* **80**, 363–387 (2016).

24. E. Karni, A mechanism for eliciting probabilities. *Econometrica* **77**, 603–606 (2009).

25. G. Malhotra, D. S. Leslie, C. J. H. Ludwig, R. Bogacz, Time-varying decision boundaries: Insights from optimality analysis. *Psychon. Bull. Rev.* **25**, 971–996 (2018).

26. R. J. Boag, R. J. Innes, N. Stevenson, G. Bahg, J. R. Busemeyer, G. E. Cox, C. Donkin, M. J. Frank, G. E. Hawkins, A. Heathcote, C. Hedge, V. Lerche, S. D. Lilburn, G. D. Logan, D. Matzke, S. Miletić, A. F. Osth, T. J. Palmeri, P. B. Sederberg, H. Singmann, P. L. Smith, T. Stafford, M. Steyvers, L. Strickland, J. S. Trueblood, K. Tsetsos, B. M. Turner, M. Usher, L. van Maanen, D. van Ravenzwaaij, J. Vandekerckhove, A. Voss, E. R. Weichart, G. Weindel, C. N. White, N. J. Evans, S. D. Brown, B. U. Forstmann, An expert guide to planning experimental tasks for evidence-accumulation modeling. *Adv. Methods Pract. Psychol. Sci.* **8**, 25152459251336127 (2025).

27. R. Ratcliff, A. Thapar, P. Gomez, G. McKoon, A diffusion model analysis of the effects of aging in the lexical-decision task. *Psychol. Aging* **19**, 278–289 (2004).

28. M. Guggenmos, Reverse engineering of metacognition. *eLife* **11**, e75420 (2022).

29. N. Haddara, D. Rahnev, The impact of feedback on perceptual decision-making and metacognition: Reduction in bias but no change in sensitivity. *Psychol. Sci.* **33**, 259–275 (2022).

30. M. Rouault, P. Dayan, S. M. Fleming, Forming global estimates of self-performance from local confidence. *Nat. Commun.* **10**, 1141 (2019).

31. R. Frey, A. Pedroni, R. Mata, J. Rieskamp, R. Hertwig, Risk preference shares the psychometric structure of major psychological traits. *Sci. Adv.* **3**, e1701381 (2017).

32. G. L. Mazza, H. L. Smyth, P. G. Bissett, J. R. Canning, I. W. Eisenberg, A. Z. Enkavi, O. Gonzalez, S. J. Kim, S. A. Metcalf, F. Muniz, W. E. Pelham III, E. A. Scherer, M. J. Valente,

H. Xie, R. A. Poldrack, L. A. Marsch, D. MacKinnon, Correlation database of 60 cross-disciplinary surveys and cognitive tasks assessing self-regulation. *J. Pers. Assess.* **103**, 238–245 (2021).

33. P. Atanasov, *Risk preferences in choices for self and others: Meta analysis and research directions* (SSRN, 2015); https://dx.doi.org/10.2139/ssrn.1682569.

34. A. Voodla, A. Uusberg, K. Desender, Metacognitive confidence and affect - two sides of the same coin? *Cogn. Emot.* **39**, 1857–1874 (2025).

35. T. Sharot, M. Rollwage, C. R. Sunstein, S. M. Fleming, Why and when beliefs change. *Perspect. Psychol. Sci.* **18**, 142–151 (2023).

36. M. Rouault, T. Seow, C. M. Gillan, S. M. Fleming, Psychiatric symptom dimensions are associated with dissociable shifts in metacognition but not task performance. *Biol. Psych.* **84**, 443–451 (2018).

37. M. Lebreton, S. Langdon, M. J. Slieker, J. S. Nooitgedacht, A. E. Goudriaan, D. Denys, R. J. van Holst, J. Luigjes, Two sides of the same coin: Monetary incentives concurrently improve and bias confidence judgments. *Sci. Adv.* **4**, eaaq0668 (2018).

38. M. Lebreton, K. Bacily, S. Palminteri, J. B. Engelmann, Contextual influence on confidence judgments in human reinforcement learning. *PLOS Comput. Biol.* **15**, e1006973 (2019).

39. C.-C. Ting, N. Salem-Garcia, S. Palminteri, J. B. Engelmann, M. Lebreton, Neural and computational underpinnings of biased confidence in human reinforcement learning. *Nat. Commun.* **14**, 6896 (2023).

40. M. Sherif, *A Study of Some Social Factors in Perception*. (Archives of Psychology, Columbia University, 1935).

41. S. E. Ash, "Effects of group pressure upon the modification and distortion of judgements," in *Groups, Leadership and Men; Research in Human Relations.* H. Guetzkow Ed. (Carnegie Press, 1951), pp. 177–190; https://psycnet.apa.org/record/1952-00803-001.

42. R. Bond, P. B. Smith, Culture and conformity: A meta-analysis of studies using Asch's (1952b, 1956) line judgment task. *Psychol. Bull.* **119**, 111–137 (1996).

43. R. Bond, Group size and conformity. *GPIR* **8**, 331–354 (2005).

44. U. Toelch, F. Panizza, H. R. Heekeren, Norm compliance affects perceptual decisions through modulation of a starting point bias. *R. Soc. Open Sci.* **5**, 171268 (2018).

45. M. Germar, V. H. Duderstadt, A. Mojzisch, Social norms shape visual appearance: Taking a closer look at the link between social norm learning and perceptual decision-making. *Cognition* **241**, 105611 (2023).

46. A. Mahmoodi, K. Ringwald, M. K. Wittmann, C. Mehring, Social context alters metacognition. PsyArXiv [Preprint] (2019). 10.31234/osf.io/ez8qw.

47. N. Trudel, P. L. Lockwood, M. F. Rushworth, M. K. Wittmann, Neural activity tracking identity and confidence in social information. *eLife* **12**, e71315 (2023).

48. L. Schooler, M. Okhan, S. Hollander, M. Gill, Y. Zoh, M. J. Crockett, H. Yu, Confidence in moral decision-making. *Collabra Psychol.* **10**, 121387 (2024).

49. M. Rouault, S. M. Fleming, Formation of global self-beliefs in the human brain. *Proc. Natl. Acad. Sci. U.S.A.* **117**, 27268–27276 (2020).

50. A. McWilliams, H. Bibby, N. Steinbeis, A. S. David, S. M. Fleming, Age-related decreases in global metacognition are independent of local metacognition and task performance. *Cognition* **235**, 105389 (2023).

51. A. Bandura, Self-efficacy: Toward a unifying theory of behavioral change. *Adv. Behav. Res. Ther.* **1**, 139–161 (1978).

52. R. Elliott, B. J. Sahakian, A. McKay, J. J. Herrod, T. W. Robbins, E. S. Paykel, Neuropsychological impairments in unipolar depression: The influence of perceived failure on subsequent performance. *Psychol. Med.* **26**, 975–989 (1996).

53. G. Zacharopoulos, N. Binetti, V. Walsh, R. Kanai, The effect of self-efficacy on visual discrimination sensitivity. *PLOS ONE* **9**, e109392 (2014).

54. H. Van Marcke, P. L. Denmat, T. Verguts, K. Desender, Manipulating prior beliefs causally induces under- and overconfidence. *Psychol. Sci.* **35**, 358–375 (2024).

55. R. van den Berg, A. Zylberberg, R. Kiani, M. N. Shadlen, D. M. Wolpert, Confidence is the bridge between multi-stage decisions. *Curr. Biol.* **26**, 3157–3168 (2016).

56. E. Schulz, S. J. Gershman, The algorithmic architecture of exploration in the human brain. *Curr. Opin. Neurobiol.* **55**, 7–14 (2019).

57. J. Gottlieb, P.-Y. Oudeyer, Towards a neuroscience of active sampling and curiosity. *Nat. Rev. Neurosci.* **19**, 758–770 (2018).

58. L. Schulz, S. M. Fleming, P. Dayan, Metacognitive computations for information search: Confidence in control. *Psychol. Rev.* **130**, 604–639 (2023).

59. P. Gerrans, *The Measure of Madness: Philosophy of Mind, Cognitive Neuroscience, and Delusional Thought* (MIT Press, 2014); 10.7551/mitpress/9780262027557.001.0001.

60. T. L. Griffiths, F. Lieder, N. D. Goodman, Rational use of cognitive resources: Levels of analysis between the computational and the algorithmic. *Top. Cogn. Sci.* **7**, 217–229 (2015).

61. C. C. Eckel, P. J. Grossman, Managing diversity by creating team identity. *J. Econ. Behav. Org.* **58**, 371–392 (2005).

62. P.-C. Bürkner, brms: An R package for bayesian multilevel models using stan. *J. Stat. Softw.* **80**, 1–28 (2017).

63. R Core Team, *R: A Language and Environment for Statistical Computing* (R Foundation for Statistical Computing, Vienna, Austria, 2021).

64. M. Shinn, N. H. Lam, J. D. Murray, A flexible framework for simulating and fitting generalized drift-diffusion models. *eLife* **9**, e56938 (2020).

65. M. Plummer, JAGS: A program for analysis of Bayesian graphical models using Gibbs sampling, in *Proceedings of the 3rd International Workshop on Distributed Statistical Computing* (Vienna, Austria, 2003), vol. 124, pp. 1–10.

66. A. Gelman, J. B. Carlin, H. S. Stern, D. B. Rubin, Eds., *Bayesian Data Analysis*, *Texts in Statistical Science* (Chapman & Hall/CRC, 2003); 10.1201/9780429258480.

67. E. Holmes, P. T. Kitterick, A. Q. Summerfield, Cueing listeners to attend to a target talker progressively improves word report as the duration of the cue-target interval lengthens to 2,000 ms. *Atten. Percept. Psychophys.* **80**, 1520–1538 (2018).

68. S. D. Brown, A. Heathcote, The simplest complete model of choice response time: Linear ballistic accumulation. *Cogn. Psychol.* **57**, 153–178 (2008).

69. L. Fontanesi, laurafontanesi/rlssm: First dev release (2021). 10.5281/zenodo.4562217.

70. A. Vehtari, A. Gelman, J. Gabry, Practical Bayesian model evaluation using leave-one-out cross-validation and WAIC. *Stat. Comput.* **27**, 1413–1432 (2017).

71. T. Sivula, M. Magnusson, A. A. Matamoros, A. Vehtari, Uncertainty in Bayesian leave-one-out cross-validation based model comparison. arXiv:2008.10296 (2025).
